# Pembrolizumab alone or in combination with chemotherapy versus chemotherapy for advanced gastric cancer: A cost‐effectiveness analysis

**DOI:** 10.1002/cam4.6389

**Published:** 2023-09-14

**Authors:** Yitian Lang, Yan Lin, Dan Li, Jiyong Liu, Xiaoyan Liu

**Affiliations:** ^1^ Department of Pharmacy, Huangpu Branch, Shanghai Ninth People's Hospital Shanghai Jiao Tong University School of Medicine Shanghai China; ^2^ Department of Pharmacy Fudan University Shanghai Cancer Center Shanghai China

**Keywords:** advanced gastric cancer, cost‐effectiveness, partitioned survival approach, pembrolizumab, the perspective of the US and China

## Abstract

**Purpose:**

The KEYNOTE‐062 trial demonstrated the efficacy and safety of pembrolizumab for advanced gastric cancer (GC). The current study evaluated the cost‐effectiveness of pembrolizumab alone or in combination with chemotherapy versus chemotherapy for advanced GC from the perspective of the United States and China. And the results will provide evidence and data support for more drug selection‐related decisions and research in the future.

**Methods:**

A partitioned survival approach with three states was created for treatment of advanced GC. The survival data were derived from KEYNOTE‐062 trial and the individual patient data were generated by a specific algorithm. We fitted 21 survival functions to each treatment arm and selected the most suitable distribution type for each one. Direct costs and utility values were collected from the published, available database. Cost, quality‐adjusted life‐years (QALYs), and incremental cost‐utility ratios (ICURs) were considered as the primary measure outcomes. One‐way and probabilistic sensitivity analyses were performed to assess the reliability of the analyses.

**Results:**

In the base‐case analysis of combined positive score (CPS) ≥1 patients, the ICUR of pembrolizumab plus chemotherapy versus chemotherapy in American and Chinese setting is $345,209/QALY and $186,802.6/QALY, respectively. And the ICUR of pembrolizumab versus chemotherapy is $473,650/QALY and $377,753/QALY in the context of the US and China, respectively. For CPS≥10 patients, the ICUR of pembrolizumab plus chemotherapy versus chemotherapy in American and Chinese setting is $483,742/QALY and $262,965/QALY, respectively. And that of pembrolizumab versus chemotherapy is $96,550/QALY and $67,896/QALY in the context of the US and China.

**Conclusion:**

Compared with chemotherapy, either pembrolizumab plus chemotherapy or pembrolizumab monotherapy is not regarded as a cost‐effective strategy for patients with CPS≥1, advanced gastric cancer in the current American and Chinese setting. But pembrolizumab monotherapy for CPS≥10 patients would become a cost‐effective option in the American setting.

## 1 INTRODUCTION

With approximately 1,033,000 new cases and about 782,000 deaths in 2018, stomach cancer ranked fifth among common malignant tumors and was the third leading cause of cancer‐related deaths globally.^1^ Stomach cancer is classified anatomically into non‐cardia gastric cancers (NCGC, gastric adenocarcinomas) and cardia gastric cancers (CGC, gastroesophageal‐junction adenocarcinomas).^2^ Gastric cancer (GC) still remains a significant health burden worldwide,^3^ especially in Asia, Latin America, central, and eastern Europe.^4^ About 80%–90% of people with GC are diagnosed at an advanced stage when they are first diagnosed, which implies that either the tumor cannot be removed through surgery or the tumor developed a recurrence or metastasis.^3^ GC in its advanced stages, however, has a poor prognosis. Usually, when human epidermal growth factor receptor 2 (HER2) is positive, clinical guidelines recommend that patients with advanced gastric cancer receive targeted therapy (trastuzumab combined with chemotherapy), and for HER2‐negative gastric cancer patients, chemotherapy and immunotherapy are recommended.^5^ Especially, several breakthroughs have been made in the program death protein 1 (PD‐1)/program death ligand 1 (PD‐L1) axis immunotherapies in recent years. It is believed that PD‐1/PD‐L1 blockers work by sensitizing people's immune systems to cancer cells in order to prevent immune escape.^6^ Numerous trials have demonstrated significant efficacy and safety of PD‐1 inhibitors. The CheckMate‐649 trial evaluated the efficacy and safety of treatment with nivolumab combined with chemotherapy. It indicated that nivolumab plus chemotherapy provided significant overall survival (OS) benefit (hazard ratio [HR] = 0.71) and progression‐free survival (PFS) benefit (HR = 0.68) versus chemotherapy regimen in patients with a PD‐L1 combined positive score (CPS) of five and more.^7^ The KEYNOTE‐059 and KEYNOTE‐061 trials showed promising results for pembrolizumab.^8^, ^9^ The KEYNOTE‐062 trial showed that pembrolizumab was not inferior to chemotherapy, with fewer adverse events observed.^10^ Therefore, the PD‐1 inhibitor appears to be a promising treatment option for the population with advanced gastric cancer, especially for those with positive expression of PD‐L1. Pembrolizumab is a humanized monoclonal IgG4 kappa anti‐human PD1 antibody. Pembrolizumab's binding to PD‐1 does not stimulate Fc receptors or activate complement, so it has no cytotoxicity.^11^ Thus, compared with chemotherapy regimen, pembrolizumab monotherapy has better safety.

For advanced or metastatic tumors, new cancer therapeutics, including immune checkpoint inhibitors, and chimeric antigen receptor T‐cell therapy, have become available. Efficacy and safety are necessary but not sufficient criteria for choosing drug therapies. Cost‐effectiveness is also crucial because high prices of these drugs have sparked debates about the degree to which they provide value.^12^, ^13^, ^14^ In healthcare decision‐making, clinicians and policymakers must consider cost‐effectiveness to allocate limited resources optimally. However, the application of pembrolizumab to the treatment of gastric cancer may cause significant economic burden. No economic studies or evidence have evaluated pembrolizumab alone or with chemotherapy for advanced gastric cancer to date, which greatly constrains future decision‐making and research on pembrolizumab selection. The above reasons motivated us to conduct this study. Thus, we performed an economic evaluation and estimated the cost‐effectiveness of pembrolizumab or pembrolizumab plus chemotherapy for population with advanced GC from the perspectives of the United States and China.

## 2 MATERIALS AND METHODS

### 2.1 Model structure

We developed a decision analytic model for pembrolizumab, pembrolizumab plus chemotherapy, and chemotherapy alone regimens to assess cost‐effectiveness from the perspective of the United States and China. A partitioned survival approach model (PartSA) was used to simulate disease survival of GC patients beyond the follow‐up period of the clinical trial. The simulated population was followed with the KEYNOTE‐062 trial,^10^ who were at least 18 years of age with histologically diagnosed with locally advanced, unresectable or metastatic GC and had not received neoadjuvant or adjuvant therapy 6 or more months before randomization. Three interventions are offered to patients in this study until disease progressed: (i) chemotherapy (38% of patients received cisplatin with fluorouracil, and the cisplatin with capecitabine regimen for 62% of patients); (ii) pembrolizumab; (iii) pembrolizumab plus chemotherapy (38.1% of patients received pembrolizumab with cisplatin and fluorouracil, and the pembrolizumab with cisplatin and capecitabine regimen for 61.9% of patients). As the disease progresses, the current treatment regimen is considered invalid and would be discontinued. Afterward, 54% of patients in the chemotherapy group, 53% in the pembrolizumab group and 47% in the pembrolizumab plus chemotherapy group received subsequent treatment anticancer regimens.^10^ In addition, the maximum administration cycle of pembrolizumab is 2 years (35 cycles).

**FIGURE 1:**
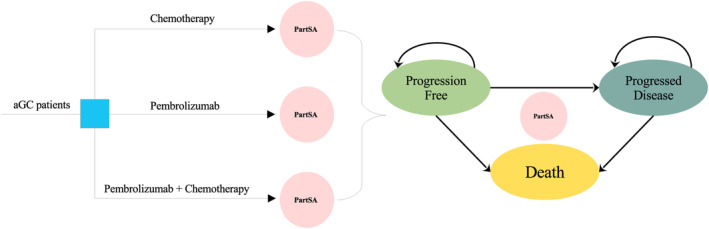
The overview of decision tree and model structure. aGC, advanced gastric cancer; PartSA, partitioned survival approach.

In the PartSA model, there are three mutually exclusive health states, including progression‐free (PF), progressed disease (PD), and death. An overview of decision tree and model structure can see Figure 1. The PF state is assumed as a default state when advanced GC patients entered the model, then moved to the PD or death state depending on survival data. Model cycles followed the KEYNOTE‐062 protocol and was set 3 weeks. Predicting long‐term outcomes requires extrapolation of limited survival data, which is necessary for a comprehensive understanding of the survival outcome. Therefore, a 10‐year horizon was selected to ensure the advanced GC population reached the death stage fully.

### 2.2 Clinical data

In the KEYNOTE‐062 trial, the timeframe of OS is around 42 months, and the PFS is 32 months. Nevertheless, the follow‐up time of the trial is not sufficient for the analysis in the setting of a 10‐year timeframe. We extrapolated the follow‐up time using algorithms developed by Guyot for predicting survival.^15^ Engauge Digitizer, a tool of digitizing figures, is used to digitize the time‐to‐survival point data of Kaplan–Meier curves for each treatment arm. A pseudo individual participant data (IPD, time‐to‐event data) was generated by algorithms and was applied to fit a range of survival functions, including Weibull, exponential, log‐normal, log‐logistic, Gompertz, Generalized Gamma, Mixture cure model, non‐Mixture cure model, and Royston–Parmar spline model, (Table S1). In general, the most appropriate survival function is determined by the Akaike information criterion (AIC).^16^ For serious adverse events (AEs), it hardly appeared in pembrolizumab group. And in the chemotherapy or pembrolizumab plus chemotherapy group, serious adverse events should be considered, which included nausea, fatigue, anemia, neutropenia, diarrhea, neutrophil count decreased, etc. The key survival parameters and incidence of serious adverse events were summarized in Table 1.

**TABLE 1:** Key clinical data.

PD‐L1 CPS expression	Parameters	Estimated values	Range	Distribution	References
CPS≥1 group	PFS: Pembrolizumab plus chemotherapy	Shape = 1.7713 Scale = 9.4858	NA	Log‐logistic	10
	PFS: Pembrolizumab	Gamma0 = −3.1179 Gamma1 = 6.5452 Gamma2 = 1.4836 Gamma3 = −1.0483	NA	Royston–Parmar spline model (2 knot)	10
	PFS: Chemotherapy	Ghape = 2.1702 Scale = 8.9861	NA	Log‐logistic	10
	OS: Pembrolizumab plus chemotherapy	Theta = 0.1174 Shape = 1.2763 Scale = 20.6033	NA	Mixture cure model (Weibull)	10
	OS: Pembrolizumab	Gamma0 = −3.7001 Gamma1 = 2.0030 Gamma2 = 0.2087 Gamma3 = −0.1942	NA	Royston–Parmar spline model (2 knot)	10
	OS: Chemotherapy	Shape = 1.8676 Scale = 15.4970	NA	Log‐logistic	10
CPS≥10 subgroup	PFS: Pembrolizumab plus chemotherapy	Meanlog = 2.2674 SDlog = 1.1422	NA	Log‐normal	10
	PFS: Pembrolizumab	Gamma0 = −2.2851 Gamma1 = 3.2239 Gamma2 = 0.3712 Gamma3 = −0.2247	NA	Royston–Parmar spline model (2 knot)	10
	PFS: Chemotherapy	Shape = 2.1568 Scale = 8.456	NA	Log‐logistic	10
	OS: Pembrolizumab plus chemotherapy	Shape = 1.3949 Scale = 17.9938	NA	Log‐logistic	10
	OS: Pembrolizumab	Meanlog = 3.0061 SDlog = 1.8325	NA	Log‐normal	10
	OS: Chemotherapy	Shape = 1.724 Scale = 15.6818	NA	Log‐logistic	10
Incidence of main Grade 3–5 AE in the chemotherapy group					
Nausea		7.4%	(5.55%–9.25%)	Beta	10
Fatigue		5.7%	(4.28%–7.13%)	Beta	10
Anemia		14.3%	(10.73%–17.88%)	Beta	10
Neutropenia		27.9%	(20.93%–34.88%)	Beta	10
Diarrhea		5.7%	(4.28%–7.13%)	Beta	10
Neutrophil count decreased		9.0%	(6.75%–11.25%)	Beta	10
Incidence of main Grade 3–5 AE in the pembrolizumab plus chemotherapy group					
Nausea		7.6%	(5.70%–9.50%)	Beta	10
Fatigue		7.6%	(5.70%–9.50%)	Beta	10
Anemia		12%	(9.00%–15.00%)	Beta	10
Neutropenia		25.2%	(18.90%–31.50%)	Beta	10
Diarrhea		4.8%	(3.60%–6.00%)	Beta	10
Neutrophil count decreased		13.6%	(10.20%–17.00%)	Beta	10

Abbreviations: AE, adverse event; CPS, combined positive score; PD‐L1, program death ligand 1; PFS, progression‐free survival; OS, overall survival.

### 2.3 Costs and utilities

A perspective of the payer was adopted in this analysis. Only the direct medical costs were included in the cost estimates and as follows: costs related to agents, administration for intravenous injection, management of serious AEs, best supportive care (BSC), and palliative care. The agent doses are in accordance with the KEYNOTE‐062 trial. The chemotherapy regimen among three regimens, cisplatin was used at a dose of 80 mg/m^2^/day administered intravenously on Day 1 of each model cycle, capecitabine (1000 mg/m^2^ twice daily administered orally on Days 1–14 of each model cycle) or fluorouracil (800 mg/m^2^/day administered intravenously on Days 1–5). In the pembrolizumab or pembrolizumab plus chemotherapy regimen, the dose of pembrolizumab was 200 mg per model cycle. The mean body surface area (BSA) of 1.85 m^2^ is used for American population.^17^ And that of 1.72 m^2^ is adopted for Chinese patients.^18^ The prices of cisplatin, fluorouracil, capecitabine, and pembrolizumab for the analysis of American setting were sourced from the Centers for Medicare and Medicaid Services (CMS).^19^ And a database of local charges for drug acquisition was used to obtain those of China.^20^ Published literature or CMS data were used to determine the costs associated with intravenous injection administration, palliative care, and BSC.^21^, ^22^, ^23^, ^24^, ^25^ Data about severe AEs were shared in the KEYNOTE‐062 trial, from which we extracted the incidences of AE that occurred. Grade 1 and 2 AEs management costs were not considered because they can be well managed, so only the costs linked to management of Grade 3–5 AEs were included. The cost data for managing AEs were sourced from open accessed database or published literatures.^23^, ^26^, ^27^, ^28^ With the Consumer Price Index (CPI) calculator, all costs for years prior to 2021 have been converted to US dollars (USD) in 2021. And the costs sourced from China were converted to USD using the average exchange rate for 2021.

In this PartSA model, each health state was assigned a health utility value based on the stage of disease progression. The health utility data from the KEYNOTE‐062 trial wasn't available, so direct data on quality‐of‐life could not be retrieved. Data that is highly relevant and robust is essential. The quality of life is assumed to be related to the progressive stage of the tumor. Therefore, the utility value for PF state was estimated to be 0.797 and 0.577 for tpatients.^29^, ^30^, ^31^, ^32^ The utility decrement for chemotherapy‐related adverse events was estimated as 0.044 based on a systematic review.^33^ More detailed inputs values are summarized in Table 2.

**TABLE 2:** Key model inputs costs, utility estimates, and other parameters.

Parameter	Distribution	The US		China	
Treatment costs		Values (range), USD	Reference	Values (range), USD	Reference
Pembrolizumab (per 100 mg)	Gamma	5230 (3922.5–5230)	19	2777.47 (2083.10–2777.47)	20
Cisplatin (per 10 mg)	Gamma	1.864 (1.40–2.33)	19	1.17 (0.88–1.46)	20
Capecitabine (per 500 mg)	Gamma	0.843 (0.632–1.054)	19	4.28 (3.21–5.35)	20
Fluorouracil (per 500 mg)	Gamma	2.129 (1.597–2.661)	19	22.26 (16.70–27.83)	20
Administration (per cycle)	Gamma	238.09 (178.57–297.61)	21	61.85 (46.39–77.31)	23
Subsequent treatment (per cycle)	Gamma	8207.25 (6155.44‐10,259.06)	22	1857.49 (1393.12–2321.86)	25
Palliative care	Gamma	11,820 (8865‐14,775)	24	2103.61 (1577.71‐2629.51)	23

AEs unit costs		Values (range), USD	Reference	Values (range), USD	Reference
Neutrophil count decreased	Gamma	51,308 (38,481–64,135)	26	105.17 (78.88–131.46)	23
Neutropenia	Gamma	51,337 (38,503–64,171)	26	528.02 (396.02–660.03)	23
Anemia	Gamma	36,264 (27,198–45,330)	26	608.35 (456.26–760.44)	23
Fatigue	Gamma	27,931 (20,948–34,914)	26	1721.3 (1291.0‐2151.6)	28
Nausea	Gamma	25,956 (19,467–32,445)	26	43.14 (32.36–53.93)	27
Diarrhea	Gamma	30,926 (23,195–38,658)	26	1006.5 (754.9–1258.1)	28

Utility estimates		Values (range)	Reference	Values (range)	Reference
PFS state	Beta	0.797 (0.598–0.996)	29	0.797 (0.598–0.996)	30, 31, 32
PD state	Beta	0.577 (0.433–0.721)	29	0.577 (0.433–0.721)	30, 31, 32

Disutility estimates		Values (range)		Reference	
Chemotherapy‐Related AEs	Beta	0.044 (0.033–0.055)		33	

Other parameters		Values (range)	Reference	Values (range)	Reference
Body surface area, m^2^	Normal	1.85 (1.49–2.21)	17	1.72 (1.50–1.90)	18

*Note*: In this table, the costs of AEs presented were paid on a per‐event basis. All costs reported for years prior to 2021 are updated to December 2021 USD using the CPI. And all costs sourced from China have been converted to US dollars using the average exchange rate for 2021 ($1 = RMB 6.4512).

Abbreviations: AEs, adverse events; CMS, Centers for Medicare and Medicaid Services; CPI Consumer Price Index; USD, US dollars.

### 2.4 Analyses

In this study, the primary simulated population for economic evaluation is PD‐L1 CPS≥1 patients. In the base‐case analysis, the incremental cost per additional life‐year (LY) gained among the regimens was assessed using the incremental cost‐effectiveness ratio (ICER). To estimate the incremental cost per additional quality‐adjusted life‐year (QALY), incremental cost‐utility ratios (ICURs) were used. The costs and QALY were discounted at 3% annually. If the ICUR is below the willingness‐to‐pay (WTP) threshold, it indicates the regimen is “cost‐effective”. Commonly, the WTP threshold that researchers select for analysis is around $100,000–150,000/QALY in the United States.^34^ Here, the threshold of $150,000/QALY was used for the setting of the United States. In China, the WTP threshold was set at three times the per capita gross domestic product A threshold of three times the average per capita gross domestic product (GDP) was used for the WTP in China (3 × per capita GDP, calculated to be $37,650 in 2021).^35^

To determine whether our results are robust and which variable had a significant impact on them, we conducted both one‐way and probabilistic sensitivity analysis (PSA) for all model inputs in the PD‐L1 CPS ≥1 population. In one‐way sensitivity analyses, the range of annual discount rate is from 0% to 8% and other inputs of that were assumed a variation by ± 25% of the base‐case value. And Monte Carlo simulations with 1000 iterations were used for PSA to examine the influence of parameter uncertainty. Each model input was sampled randomly from its probability distribution during 1000 simulations. Gamma distribution was used for cost inputs, and Beta distribution was used for health utility inputs and AE incidence inputs.^36^ The cost‐effectiveness acceptability curve (CEAC) was developed to clearly present the likelihood that treatment strategies were cost‐effective at a range of WTP thresholds.

Furthermore, considering that PD‐L1 expression may have a certain impact on survival outcomes, it will inevitably have a certain impact on economic results. Therefore, this study also performed a base‐case analysis for subgroup patients with PD‐L1 CPS of 10 and higher to evaluate the cost‐effectiveness of receiving pembrolizumab or pembrolizumab plus chemotherapy in advanced gastric cancer with high PD‐L1 expression. All models in this analysis, including partitioned survival model and the cost‐effectiveness model, were programmed through R.

## 3 RESULTS

### 3.1 Replicated Kaplan–Meier survival curve and the predicted survival curve

**FIGURE 2:**
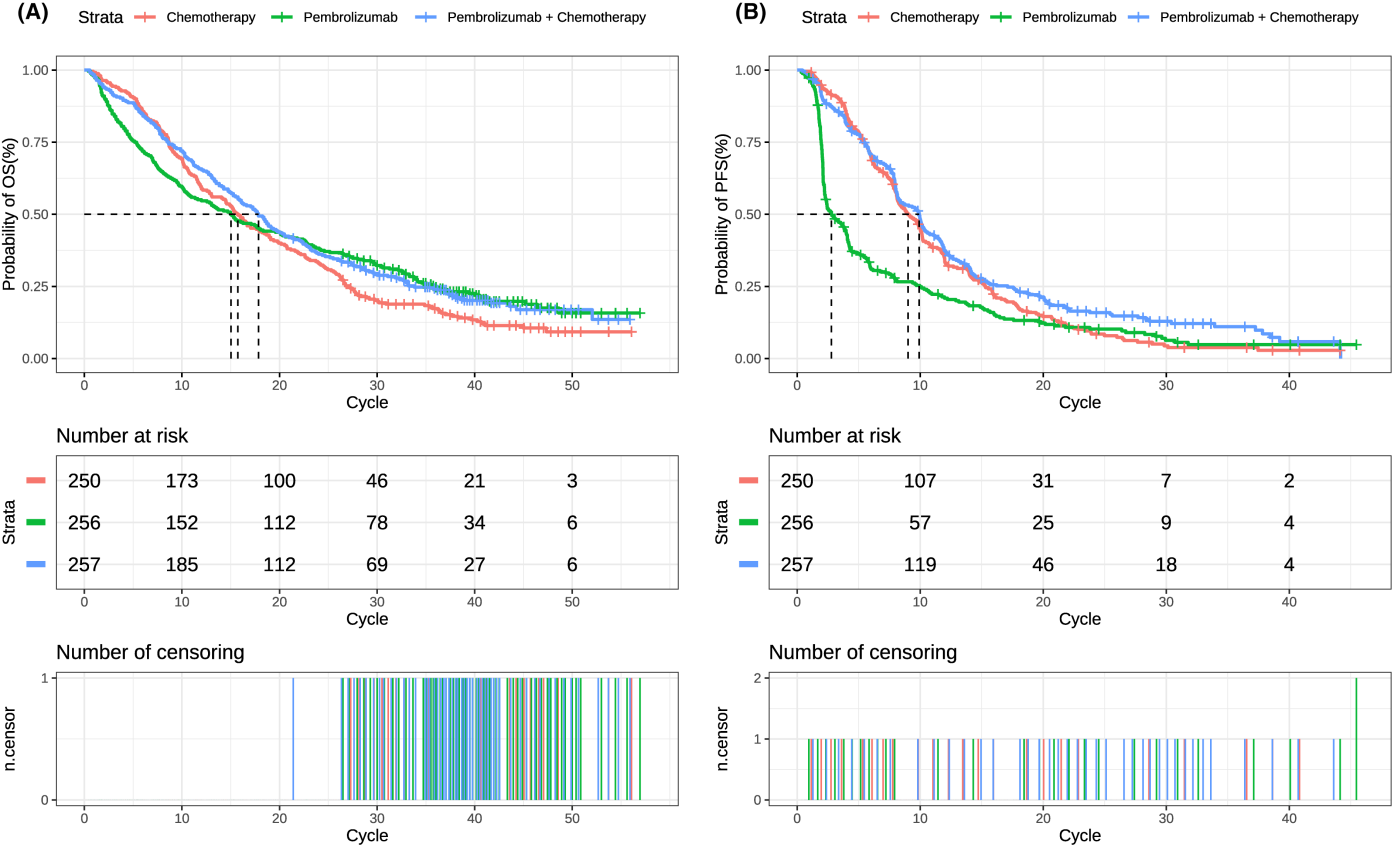
Replicated Kaplan–Meier survival curve in different regimens. (A) Output of OS curve; (B) output of PFS curve. Each cycle of the *x*‐axis is 3 weeks. PFS, progression‐free survival; OS, overall survival.

As shown in Figure 2, replicated Kaplan–Meier survival curves for PD‐L1 CPS ≥1 population were generated. Additionally, the predicted PFS and OS curves of every treatment regimen also were simulated (Figure S1). The selected distribution and parameter value of each arm can be seen in Table 1. All estimated parameters and AIC value from each survival model were listed in Table S1.

### 3.2 Base‐case analysis

All base‐case results were summarized in Table 3.

**TABLE 3:** Results of the base‐case analysis for CPS ≥1 and CPS ≥10 patients from the perspective of the United States and China.

PD‐L1 expression	Country	Regimen	LYs	QALYs	Cost, US$	ICER($/LY)	ICUR($/QALY)
CPS≥1	The United States	Chemotherapy	1.4008	0.8741	72,078.0	–	–
		Pembrolizumab	1.5552	0.9714	118,164.2	298,486	473,650.6
		Pembrolizumab plus chemotherapy	2.1548	1.2469	200,772.2	170,682	345,209.8
	China	Chemotherapy	1.4008	0.8741	13,817.6	–	–
		Pembrolizumab	1.5552	0.9714	50,573.0	238,053	377,753.3
		Pembrolizumab plus chemotherapy	2.1548	1.2469	83,457.6	92,361	186,802.6
CPS≥10	The United States	Chemotherapy	1.4981	0.9108	71,845.1	–	–
		Pembrolizumab	2.8109	1.6744	145,571.2	56,159.4	96,550.7
		Pembrolizumab plus chemotherapy	1.9615	1.1959	209,760.2	297,615.7	483,742.9
	China	Chemotherapy	1.4981	0.9108	13,563.2	–	–
		Pembrolizumab	2.8109	1.6744	65,408.7	39,492.3	67,896.1
		Pembrolizumab plus chemotherapy	1.9615	1.1959	88,534.5	161,785.3	262,964.9

Abbreviations: CPS, Combined Positive Score; ICER, incremental cost‐effectiveness ratio; ICUR, incremental cost‐utility ratio; LY life‐year; PD‐L1, program death ligand 1; QALY, quality‐adjusted life‐year.

### 3.3 Scenario 1: The context of the USA

In the US context, population with advanced PD‐L1 CPS≥1 GC received pembrolizumab plus chemotherapy regimen gained 2.1548 LY, 1.2469 QALYs and expended $200,772. And receiving pembrolizumab regimen resulted in 1.5552 LY, 0.9714 QALYs gained and $118,164 expended. And the chemotherapy regimen resulted in 1.4008 LY, 0.8741 QALY and $72,078 expended. Compared with the chemotherapy regimen, the pembrolizumab plus chemotherapy regimen increased the overall cost by $128,694, pembrolizumab monotherapy increased by $46,086.2. For effectiveness, pembrolizumab plus chemotherapy regimen showed an increase of 0.754 LY, 0.3728 QALYs compared with chemotherapy regimen, and the increase was 0.1544 LY, 0.0973 QALYs in the pembrolizumab monotherapy regimen. The ICER and ICUR of pembrolizumab plus chemotherapy versus chemotherapy is $170,682 /LY and $345,209.8/QALY, respectively. The ICER and ICUR of pembrolizumab compared with chemotherapy is $298,486/LY and $473,650.6/QALY, respectively. In the higher PD‐L1 expression subgroup of CPS≥10, pembrolizumab plus chemotherapy regimen gained 1.9615 LY, 1.1959 QALYs and expended $209,760. And receiving pembrolizumab monotherapy resulted in 2.8109 LY, 1.6744 QALYs gained and $145,571 expended. the chemotherapy regimen resulted in 1.4981 LY, 0.9108 QALY and $71,845 expended. The ICER and ICUR of pembrolizumab plus chemotherapy versus chemotherapy is $297,615/LY and $483,742.9/QALY, respectively. The ICER and ICUR of pembrolizumab compared with chemotherapy is $56,159/LY and $96,550.7/QALY, respectively.

### 3.4 Scenario 2: The context of China

In the context of China, population with advanced PD‐L1 CPS≥1 GC received pembrolizumab plus chemotherapy regimen gained 2.1548 LY, 1.2469 QALYs and $83,457 in expenditures. And receiving pembrolizumab regimen brought about 1.5552 LY, 0.9714 QALYs and $50,573 in expenditures. And the chemotherapy regimen resulted in 1.4008 LY, 0.8741 QALY and expended $13,817. In comparison with the chemotherapy regimen, the pembrolizumab plus chemotherapy regimen increased the overall cost by $69,640, whereas the increase of pembrolizumab monotherapy is $36,755. As far as effectiveness is concerned, pembrolizumab plus chemotherapy regimen presented a rise of 0.754 LY and 0.3728 QALY compared with chemotherapy regimen, and the increase was 0.1544 LY, 0.0973 QALYs in the pembrolizumab monotherapy regimen. The ICER and ICUR of pembrolizumab plus chemotherapy compared with chemotherapy is $92,361 /LY and $186,802.6/QALY, respectively. The ICER and ICUR of pembrolizumab versus chemotherapy is $238,053 /LY and $377,753.3/QALY, respectively. Among patients with high PD‐L1 expression of CPS≥10, the ICER and ICUR of pembrolizumab plus chemotherapy versus chemotherapy were $161,785/LY and $262,964 /QALY, respectively. The ICER and ICUR of pembrolizumab compared with chemotherapy were $39,492/LY and $67,896/QALY, respectively.

### 3.5 One‐way sensitivity analysis

One‐way sensitivity analysis outcomes are displayed in tornado diagrams to demonstrate the sensitivity of outputs to model inputs.

### 3.6 Scenario 1: American setting

**FIGURE 3:**
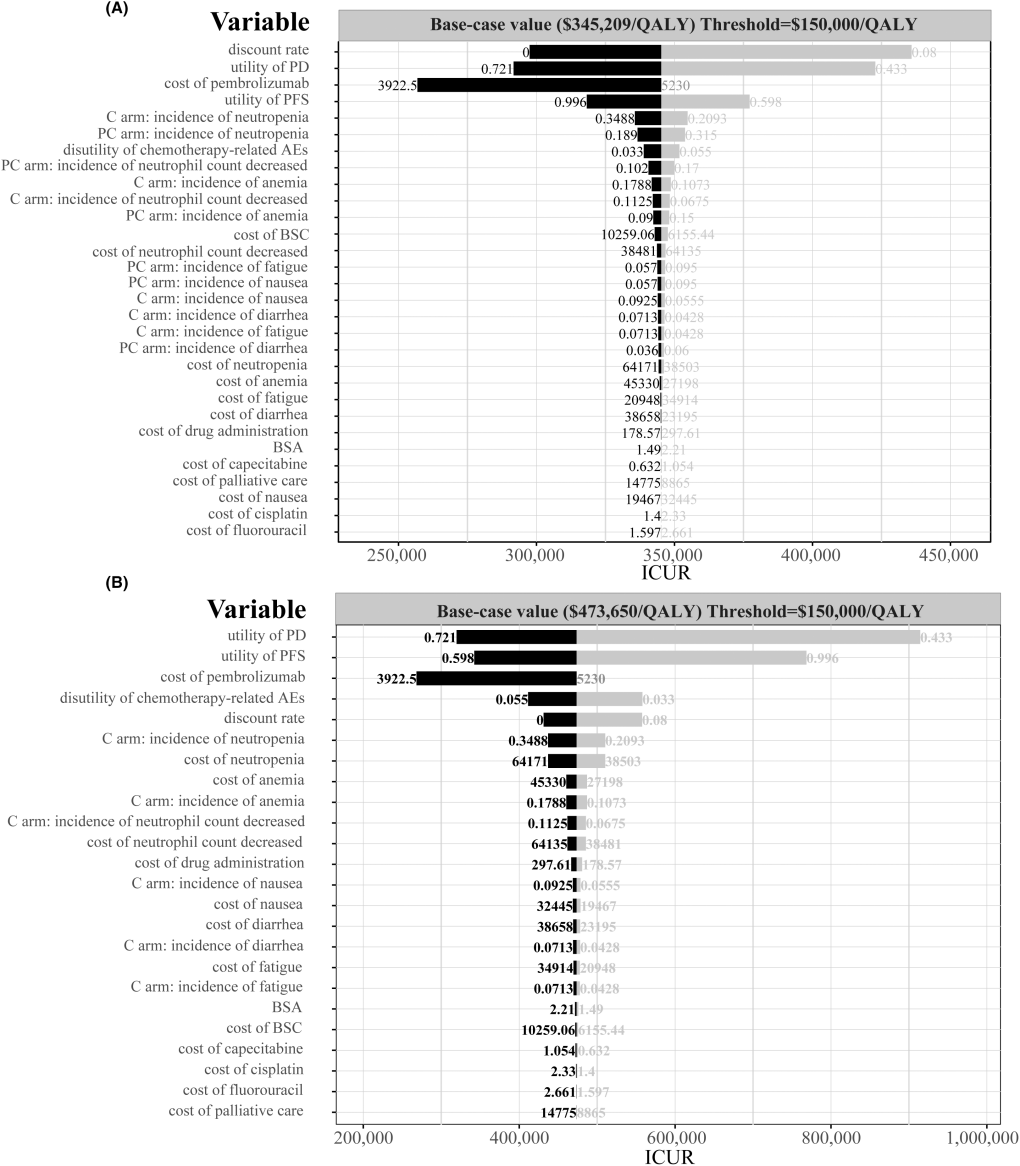
Tornado diagram of one‐way sensitivity analysis. (A) The output of PC versus C in the American setting. (B) The output of P versus C in the American setting. AEs, adverse events; BSA, body surface area; BSC, best supportive care; C, chemotherapy; ICUR, Incremental cost‐utility ratio; P, pembrolizumab; PC, pembrolizumab plus chemotherapy; PD, progressed disease; PFS, progression‐free survival; QALY, quality‐adjusted life‐year.

We can find that, from the diagram of tornado (Figure 3A), discount rate, the utility of PD, and the price of pembrolizumab were the key driving factors that had a significant impact on ICUR between PC and C regimen. The range for the one‐way sensitivity analysis was from $256,884.3/QALY to $435,888.1/QALY. The tornado diagram for P versus C regimen (Figure 3B) showed that the utility of PD or PFS, the price of pembrolizumab and disutility of chemotherapy‐related AEs could yield significant effects on the ICUR. The range was from $268,211.0/QALY to $914,493.0/QALY. One of the most potential variables to lower the ICUR is the price of pembrolizumab. The impact of other inputs on the ICUR was not prominent.

### 3.7 Scenario 2: Chinese setting

In the Chinese context, the key variables affecting ICUR were similar to those of the American setting. In the comparison of PC versus C, discount rate, utility of PD, cost of pembrolizumab, and utility of PFS were the main factors influencing ICUR (Figure S2). The boundary of ICUR across PC and C regimens ranged from $139,896/QALY to $235,927.3/QALY. In the comparison between P and C, the range was from $254,916.5/QALY to $729,343.6/QALY. The utility of PD, price of pembrolizumab and the utility of PFS are the most potential variables to lower the ICUR. In addition, the disutility of chemotherapy‐related AEs and discount rate also contributes to the reduction of ICUR. Other parameters had an inconspicuous impact on the ICUR.

### 3.8 Probabilistic sensitivity analysis

**FIGURE 4:**
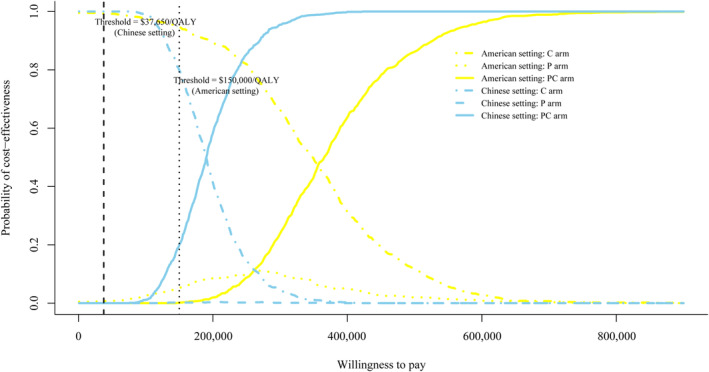
Cost‐effectiveness acceptable curve. The *y*‐axis indicates the likelihood that a regimen is cost‐effective across the willingness‐to‐pay threshold (*x*‐axis*)*. C, chemotherapy; P, pembrolizumab; PC, pembrolizumab plus chemotherapy; QALY, quality‐adjusted life‐year.

To assess the likelihood of each treatment regimen would be regarded as cost‐effective at a range of thresholds, a CEAC was created (Figure 4).

### 3.9 Scenario 1: The context of the USA

The CEAC showed pembrolizumab plus chemotherapy regimen was 0.1% cost‐effective with a threshold of $150,000/QALY, and that the pembrolizumab regimen was 5.1% cost‐effective at the same threshold. Because the price of pembrolizumab was a potential variable for lowering the ICUR, an additional probabilistic sensitivity analyses of adjusting the price of pembrolizumab to 75%, 50%, and 25% of its price were conducted. The further CEAC could be seen in Figure S3A. The likelihood of cost‐effectiveness for pembrolizumab plus chemotherapy was 0.4%, 5.3%, and 33.8%, respectively, when the price of pembrolizumab was reduced to 75%, 50%, and 25% of the base value. Similarly, the likelihood of cost‐effectiveness for pembrolizumab alone was 24.3%, 69.9%, and 66.1%, respectively, under the same price reductions. More outputs were summarized in Table S2.

### 3.10 Scenario 2: The context of China

Similar to what we have seen in American settings, at the threshold of $37,650/QALY, both pembrolizumab plus chemotherapy and pembrolizumab monotherapy regimen have almost zero likelihood of being cost‐effective. Figure S3B also showed the changing trend of the probability when the price of pembrolizumab was reduced to 75%, 50%, and 25%. The probabilities of cost‐effectiveness were almost unaffected in the setting of 75% and 50% of the base value. Nevertheless, the likelihood was 28.1% when the price is 27.3% of the base value in the pembrolizumab regimen. And the probability that pembrolizumab plus chemotherapy regimen would be deemed as cost‐effective was 12.7%.

## 4 DISCUSSION

To our knowledge, our analysis is the first to examine the cost‐effectiveness of pembrolizumab alone or in combination with chemotherapy versus chemotherapy alone for patients with CPS of one and more, advanced gastric cancer. The results of base‐case analysis showed that the ICUR of pembrolizumab plus chemotherapy versus chemotherapy was $345,209/QALY (American setting) and $186,802/QALY (Chinese setting), respectively. And the ICUR of pembrolizumab versus chemotherapy was $473,650/QALY (American setting) and $377,753/QALY (Chinese setting), respectively. The ICUR values significantly exceeded the WTP threshold, which made us clearly realize the pembrolizumab alone or pembrolizumab plus chemotherapy regimens were not cost‐effective options compared with chemotherapy strategy whether in American setting or Chinese setting. One‐way sensitivity analysis indicated that the price of pembrolizumab were the most influential to the ICUR‐lowering. Therein, the greater potential to lower the ICUR existed in the variable of the price of pembrolizumab. In order to investigate the cost‐effectiveness driven by the price of pembrolizumab, further probabilistic sensitivity analyses were created. We made several scenarios assumptions (75%, 50%, and 25% of the base‐case price) to explore the changing trends in likelihood of cost‐effective. As the price of pembrolizumab decreases, pembrolizumab monotherapy regimen became more cost‐effective, especially in the setting of the United States. Although we found that pembrolizumab plus chemotherapy regimen achieved more QALY and lower ICUR in the base‐case analysis, it may be related to the failure of the monotherapy regimen of pembrolizumab to delay the progression of the disease according to the Kaplan–Meier estimates of progression‐free survival of KEYNOTE‐062 trial (HR 1.66; 95% CI, 1.37–2.01). Compared with the chemotherapy regimen, we cannot ignore the lower incidence of adverse events and the future potential of survival benefits brought by pembrolizumab monotherapy.

Currently, there is still a lack of economic evaluation of pembrolizumab alone or with chemotherapy for gastric cancer to compare with our analysis. We retrieved some studies on other immune checkpoint inhibitors, and similar conclusion has been drawn from these studies. Jiang et al. calculated the ICUR of nivolumab plus chemotherapy compared with chemotherapy alone in the all patients group with gastric cancer was $191,266/QALY from the Chinese healthcare system perspective, exceeding the WTP threshold of $33,436/QALY.^37^ And the tornado diagram from the study of Jiang et al. also showed the cost of nivolumab had significant effect on the ICUR. Although this is a study on cost‐effectiveness of nivolumab rather than pembrolizumab, the sensitive inputs and result were almost consistent with our analysis outcomes of pembrolizumab plus chemotherapy versus chemotherapy in the patients with CPS≥1, advanced gastric cancer (ICUR: $186,802/QALY, exceeding the WTP threshold of $37,650/QALY). It suggested that, owing to the high pricing, the two PD‐1 inhibitors are unlikely to be cost‐effective options under the current economic context in China. Another study from the Japanese public healthcare system perspective revealed that the ICER of nivolumab versus trifluridine/tipiracil was $294,113/QALY, exceeding the threshold value of $68,182/QALY.^38^ These studies indicated the utility of disease stage and the cost of PD‐1 inhibitor were the key variables that limited the cost‐effectiveness of PD‐1 inhibitor. Compared with chemotherapy for gastric cancer, the contribution of PD‐1 inhibitor to improving the QALY was not significant, and it also consumes more costs in drugs. The high cost of PD‐1 inhibitors for advanced gastric cancer imposes a substantial burden on society, health care systems, and patients in the United States, China, and other countries. This analysis informs drug selection for patients with advanced or metastatic gastric cancer and health insurance policy development in China and similar settings.

Considering the conclusion that pembrolizumab is not the preferred option is based on patients whose PD‐L1 expresses CPS≥1 in this study. In order to determine whether the analysis results are related to the degree of PD‐L1 expression, we conducted additional base‐case analyses based on PD‐L1 CPS≥10 patients. However, the conclusions changed greatly. In the CPS≥10 patients, pembrolizumab monotherapy became a cost‐effective regimen compared with chemotherapy regimen in the context of the US (ICUR = $96,550.7/QALY, WTP threshold = $150,000/QALY). Although in the Chinese setting, pembrolizumab is not still a preferred option, the ICUR value was significantly lower than that in the CPS≥1 population ($67,896/QALY vs. $377,753.3/QALY, WTP threshold = $37,650/QALY). According to the results in patients with high expression of PD‐L1, we are more certain that we cannot deny the potential of pembrolizumab monotherapy. In other words, pembrolizumab monotherapy could be approved for use in advanced gastric cancer people with high PD‐L1 expression (CPS≥10) in the future.

There are several potential weaknesses in our analysis. Firstly, the survival data was extracted from KEYNOTE‐062 trial, in which patients from Europe, North America or Australia accounted for more than half of the proportion. Asian patients made up around one‐fourth. Nevertheless, our study analyzed the survival data of overall patients regardless of whether in the setting of the US or China, the survival benefit could be affected inevitably by race. Secondly, analysis results using a range of distributions to extrapolate the survival outcomes beyond the follow‐up timeframe may result in uncertainty compared with the analysis results of follow‐up period. Although it could weaken the robustness, our sensitivity analyses covered a wide range of all variables. Through the use of the modeling techniques, predicting results are possible for this project. Thirdly, in our study, the composition of the chemotherapy regimen was based on cisplatin plus fluorouracil or capecitabine, and may not represent all chemotherapy regimens. There are ongoing studies on chemotherapy regimens, such as CAPEOX or FOLFOX, whose therapeutic efficacy may differ from the chemotherapy regimen selected in our study. As evidence continues to be updated, more research is needed in the future to continuously supplement and provide more timely references. Lastly, no reports about utility were available in the KEYNOTE‐062 trial, the direct utility data associated with health could not be obtained. Therefore, we had to extract utility data from published studies.

Beyond the limitations of our analysis, the highlights should not be ignored. As part of our initial selection of models, we considered both a Markov model and a partitioned survival model. To reduce the uncertainty caused by the hypothesis and make use of the unique advantages of the partitioned survival model for the field of cancer, we ultimately chose the PartSA model. Using the survival curve as a data source, the probability of transitioning from PF or PD to death can be calculated directly from the survival cohort proportion through the PartSA model, hence lowering hypothesis uncertainty. Furthermore, the emergence of immune‐oncology therapies with their distinct characteristics, such as delayed onset of treatment effects and potential for long‐term survival, may increase the complexity of survival analysis. Many studies have thus suggested the incorporation of more appropriate fitting methods of immuno‐oncology drugs in economic evaluations.^39^, ^40^ In the long‐term survival simulation of this study, the survival curves of each regimen were fitted to 21 survival functions, including standard parametric model, mixture cure model, non‐mixture cure model and Royston–Parmar spline model. These survival functions offer the possibility of more flexible linear extrapolation, which further improves the accuracy of the survival outcomes of this analysis.

Hopefully this analysis will be of assistance to health decision‐makers. As the evidence is updated, more relevant studies are expected to be published to continue to strengthen the credibility of evidence.

## 5 CONCLUSIONS

This study firstly performed a long‐term survival model prediction and economic evaluation of pembrolizumab alone or with chemotherapy for advanced PD‐L1 CPS≥1 and CPS≥10 gastric cancer from the perspective of both the US and China. This gives evidence and data support for future choices on whether pembrolizumab is appropriate for advanced gastric cancer therapy in the United States or China, taking into account economic aspects and health insurance policies. For PD‐L1 CPS≥1 patients, pembrolizumab alone or with chemotherapy is unlikely to be a cost‐effective option whether in the USA or China. the price of pembrolizumab is one of key factor to limit the cost‐effectiveness. Among the Higher PD‐L1 patients of CPS≥10, pembrolizumab monotherapy regimen was recommended as a cost‐effective option in the context of the US. Although this conclusion does not apply to China, this analysis may guide the selection of drugs for patients with higher PD‐L1 expression gastric cancer and the development of health insurance policies in China or similar settings. And it is expected to be a cost‐effective regimen with the support of pharmaceutical policy or healthcare insurance in the future.

## ETHICS STATEMENT

This study is based on a literature review and modeling techniques, so it does not require the approval by an institutional research ethics board.

## AUTHOR CONTRIBUTIONS

**Yitian Lang:** Formal analysis (lead); methodology (lead); project administration (lead); writing – original draft (lead). **Yan Lin:** Validation (equal). **Dan Li:** Validation (equal). **liu jiyong:** Conceptualization (equal); supervision (equal); validation (equal). **Xiaoyan Liu:** Conceptualization (lead); funding acquisition (lead); resources (lead); supervision (lead).

## FUNDING INFORMATION

This work was supported by authors' organization and the Wu Jieping Medical Foundation (grant number 320.6750.2021‐10‐28).

## CONFLICT OF INTEREST STATEMENT

All authors have no conflicts of interest that are directly relevant to the content of this article.

## Supporting information


Data S1.

## Data Availability

All data generated or analyzed during this study are included in this article/Supplementary Material.
