# Supplementary material for: Pembrolizumab alone or in combination with chemotherapy versus chemotherapy for advanced gastric cancer: A cost‐effectiveness analysis
Source: Cancer Med. 2023 Sep 14;12(18):18447–59. doi: 10.1002/cam4.6389 (PMC10557869; doi:10.1002/cam4.6389)
Supplement: Supplementary file 1 — Data S1. [file CAM4-12-18447-s001.docx]

| **Supplemental Table 1. Estimated Parameters and AIC Value from Each Survival Model** | | | | | | | | | | | | |
| --- | --- | --- | --- | --- | --- | --- | --- | --- | --- | --- | --- | --- |
| **Strategies** | Distribution | Parameter | OS | | | | | PFS | | | | |
|  |  |  | Est | L95% | U95% | SE | AIC | Est | L95% | U95% | SE | AIC |
| **Chemotherapy group, CPS≥1** | Log-logistic | shape | 1.8676 | 1.6713 | 2.0869 | 0.1058 | 1746.1091 | 2.1702 | 1.9465 | 2.4196 | 0.1205 | 1513.7133 |
|  |  | scale | 15.497 | 13.8135 | 17.3856 | 0.9093 |  | 8.9861 | 8.1286 | 9.9341 | 0.4598 |  |
|  | Weibull | shape | 1.3004 | 1.1668 | 1.4492 | 0.0719 | 1754.3182 | 1.4069 | 1.2733 | 1.5545 | 0.0716 | 1527.363 |
|  |  | scale | 22.4424 | 20.2323 | 24.894 | 1.1871 |  | 12.7422 | 11.5782 | 14.0233 | 0.6228 |  |
|  | Exponential | rate | 0.046 | 0.0403 | 0.0525 | 0.0031 | 1772.3234 | 0.0834 | 0.0732 | 0.0951 | 0.0056 | 1562.9335 |
|  | Log-normal | meanlog | 2.7169 | 2.5967 | 2.837 | 0.0613 | 1753.4163 | 2.1735 | 2.0695 | 2.2774 | 0.053 | 1519.0297 |
|  |  | Sdlog | 0.9536 | 0.8654 | 1.0507 | 0.0472 |  | 0.822 | 0.7485 | 0.9026 | 0.0393 |  |
|  | Gompertz | shape | 0.0148 | 0.0035 | 0.026 | 0.0057 | 1768.0311 | 0.0281 | 0.0129 | 0.0434 | 0.0078 | 1553.1383 |
|  |  | rate | 0.037 | 0.0297 | 0.0462 | 0.0042 |  | 0.0646 | 0.0529 | 0.079 | 0.0066 |  |
|  | Generalized Gamma | mu | 2.9082 | 2.7271 | 3.0894 | 0.0924 | 1748.802 | 2.3152 | 2.1594 | 2.471 | 0.0795 | 1515.75 |
|  |  | sigma | 0.8689 | 0.765 | 0.9868 | 0.0564 |  | 0.7791 | 0.7005 | 0.8664 | 0.0423 |  |
|  |  | Q | 0.4654 | 0.105 | 0.8258 | 0.1839 |  | 0.3715 | 0.052 | 0.691 | 0.163 |  |
|  | Royston/Parmar spline model (0 knot) | gamma0 | -4.0454 | -4.5242 | -3.5665 | 0.2443 | 1754.3182 | -3.5804 | -3.991 | -3.1699 | 0.2095 | 1527.363 |
|  |  | gamma1 | 1.3004 | 1.1595 | 1.4412 | 0.0719 |  | 1.4069 | 1.2665 | 1.5473 | 0.0716 |  |
|  | Royston/Parmar spline model (1 knot) | gamma0 | -5.095 | -6.0782 | -4.1118 | 0.5016 | 1748.8163 | -4.7152 | -5.5363 | -3.8941 | 0.4189 | 1515.7608 |
|  |  | gamma1 | 2.0758 | 1.4542 | 2.6975 | 0.3172 |  | 2.4927 | 1.8393 | 3.1461 | 0.3334 |  |
|  |  | gamma2 | 0.0752 | 0.0184 | 0.1321 | 0.029 |  | 0.0991 | 0.0429 | 0.1553 | 0.0287 |  |
|  | Royston/Parmar spline model (2 knot) | gamma0 | -4.8487 | -5.9204 | -3.777 | 0.5468 | 1750.1293 | -4.471 | -5.3531 | -3.5888 | 0.4501 | 1516.4715 |
|  |  | gamma1 | 1.7868 | 0.9232 | 2.6505 | 0.4407 |  | 2.0671 | 1.1001 | 3.0342 | 0.4934 |  |
|  |  | gamma2 | -0.0976 | -0.3901 | 0.195 | 0.1493 |  | -0.1531 | -0.5025 | 0.1962 | 0.1782 |  |
|  |  | gamma3 | 0.2186 | -0.1649 | 0.6022 | 0.1957 |  | 0.2812 | -0.1135 | 0.6759 | 0.2014 |  |
|  | Mixture cure model (Weibull) | theta | 0.0815 | 0.0434 | 0.1479 | NA | 1748.2908 | 0.0297 | 0.012 | 0.0717 | NA | 1518.2472 |
|  |  | shape | 1.4758 | 1.3023 | 1.6725 | 0.0942 |  | 1.5496 | 1.3939 | 1.7226 | 0.0837 |  |
|  |  | scale | 19.1294 | 17.0154 | 21.506 | 1.143 |  | 11.8702 | 10.8263 | 13.0147 | 0.5575 |  |
|  | Mixture cure model (Log-logistic) | theta | 0.0004 | 0 | 1 | NA | 1748.1244 | 0.0001 | 0 | 1 | NA | 1515.7232 |
|  |  | shape | 1.8683 | 1.6708 | 2.089 | 0.1065 |  | 2.1703 | 1.9463 | 2.42 | 0.1206 |  |
|  |  | scale | 15.4892 | 13.7975 | 17.3883 | 0.914 |  | 8.9855 | 8.1276 | 9.934 | 0.4601 |  |
|  | Mixture cure model (Exponential) | theta | 0.0001 | 0 | 1 | NA | 1774.3275 | 0.0001 | 0 | 1 | NA | 1564.94 |
|  |  | rate | 0.046 | 0.0403 | 0.0526 | 0.0031 |  | 0.0834 | 0.0732 | 0.0951 | 0.0056 |  |
|  | Mixture cure model (Log-normal) | theta | 0.0003 | 0 | 1 | NA | 1755.435 | 0.0014 | 0 | 0.8897 | NA | 1521.1164 |
|  |  | meanlog | 2.7163 | 2.5958 | 2.8369 | 0.0615 |  | 2.1714 | 2.0658 | 2.2771 | 0.0539 |  |
|  |  | SDlog | 0.9533 | 0.8649 | 1.0507 | 0.0473 |  | 0.8201 | 0.745 | 0.9027 | 0.0402 |  |
|  | Mixture cure model (Gompertz) | theta | 0.0943 | 0.0575 | 0.151 | NA | 1760.0187 | 0.0306 | 0.013 | 0.0703 | NA | 1540.8464 |
|  |  | shape | 0.0411 | 0.0257 | 0.0565 | 0.0079 |  | 0.0578 | 0.0394 | 0.0761 | 0.0094 |  |
|  |  | rate | 0.0334 | 0.0262 | 0.0426 | 0.0041 |  | 0.0559 | 0.0449 | 0.0696 | 0.0062 |  |
|  | Mixture cure model (Generalized Gamma) | theta | 0.0632 | 0.0197 | 0.1850 | NA | 1749.069 | 0.0225 | 0.0060 | 0.0811 | NA | 1515.451 |
|  |  | mu | 2.8888 | 2.7216 | 3.0559 | 0.0853 |  | 2.3402 | 2.1875 | 2.4929 | 0.0779 |  |
|  |  | sigma | 0.7495 | 0.5965 | 0.9416 | 0.0873 |  | 0.7140 | 0.6181 | 0.8248 | 0.0526 |  |
|  |  | Q | 0.7194 | 0.2269 | 1.2118 | 0.2512 |  | 0.5642 | 0.1790 | 0.9495 | 0.1966 |  |
|  | Non-Mixture cure model (Weibull) | theta | 0.0562 | 0.0173 | 0.1671 | NA | 1747.3086 | 0.0237 | 0.0066 | 0.0808 | NA | 1514.7149 |
|  |  | shape | 1.6018 | 1.387 | 1.8498 | 0.1176 |  | 1.7494 | 1.5479 | 1.9772 | 0.1093 |  |
|  |  | scale | 35.6018 | 24.109 | 52.5731 | 7.0807 |  | 23.1396 | 17.3247 | 30.9063 | 3.4169 |  |
|  | Non-Mixture cure model (Log-logistic) | theta | 0.0153 | 0.0016 | 0.132 | NA | 1747.2475 | 0.0068 | 0.0007 | 0.0612 | NA | 1514.2523 |
|  |  | shape | 1.653 | 1.3967 | 1.9562 | 0.1421 |  | 1.8368 | 1.5835 | 2.1307 | 0.1391 |  |
|  |  | scale | 42.0054 | 24.0816 | 73.2698 | 11.9235 |  | 24.9716 | 16.4169 | 37.9841 | 5.3439 |  |
|  | Non-Mixture cure model (Exponential) | theta | 0 | 0 | 1 | NA | 1774.8222 | 0 | 0 | 1 | NA | 1565.5714 |
|  |  | rate | 0.0006 | 0 | 0.0091 | 0.0008 |  | 0.0008 | 0.0001 | 0.009 | 0.001 |  |
|  | Non-Mixture cure model (Log-normal) | theta | 0 | 0 | 1 | NA | 1748.5356 | 0 | 0 | 0.9934 | NA | 1514.9299 |
|  |  | meanlog | 5.7519 | 2.8729 | 8.6308 | 1.4689 |  | 4.449 | 2.7997 | 6.0983 | 0.8415 |  |
|  |  | SDlog | 1.6844 | 1.1338 | 2.5025 | 0.3402 |  | 1.3815 | 1.0117 | 1.8864 | 0.2196 |  |
|  | Non-Mixture cure model (Gompertz) | theta | 0.0925 | 0.0556 | 0.1499 | NA | 1758.071 | 0.0289 | 0.0125 | 0.0656 | NA | 1536.0444 |
|  |  | shape | 0.0623 | 0.045 | 0.0797 | 0.0088 |  | 0.095 | 0.0734 | 0.1165 | 0.011 |  |
|  |  | rate | 0.0118 | 0.0087 | 0.0159 | 0.0018 |  | 0.0137 | 0.0099 | 0.0189 | 0.0023 |  |
|  | Non-Mixture cure model (Generalized Gamma) | theta | 0.0366 | 0.0012 | 0.5495 | NA | 1749.162 | 0.0099 | 0.0002 | 0.3509 | NA | 1515.777 |
|  |  | mu | 3.6888 | 2.7438 | 4.6337 | 0.4821 |  | 3.3045 | 2.5169 | 4.0920 | 0.4018 |  |
|  |  | sigma | 0.7250 | 0.3260 | 1.6119 | 0.2956 |  | 0.7550 | 0.3901 | 1.4612 | 0.2544 |  |
|  |  | Q | 0.8003 | -0.1754 | 1.7760 | 0.4978 |  | 0.6157 | -0.1721 | 1.4036 | 0.4020 |  |
| **Pembrolizumab group, CPS≥1** | Log-logistic | shape | 1.2055 | 1.0755 | 1.3512 | 0.0702 | 1686.9978 | 1.5726 | 1.412 | 1.7515 | 0.0864 | 1321.7867 |
|  |  | scale | 14.3867 | 12.0256 | 17.2115 | 1.3159 |  | 3.8268 | 3.3346 | 4.3917 | 0.2688 |  |
|  | Weibull | shape | 0.8975 | 0.7986 | 1.0086 | 0.0535 | 1694.4677 | 0.8734 | 0.7934 | 0.9615 | 0.0428 | 1395.2159 |
|  |  | scale | 24.6553 | 21.1322 | 28.7657 | 1.9397 |  | 7.4862 | 6.4246 | 8.7231 | 0.5841 |  |
|  | Exponential | rate | 0.0402 | 0.035 | 0.0462 | 0.0028 | 1695.9166 | 0.1263 | 0.1109 | 0.1438 | 0.0084 | 1401.4477 |
|  | Log-normal | meanlog | 2.6547 | 2.4785 | 2.8309 | 0.0899 | 1679.482 | 1.4543 | 1.3174 | 1.5912 | 0.0698 | 1319.9812 |
|  |  | Sdlog | 1.389 | 1.2534 | 1.5392 | 0.0728 |  | 1.1004 | 1.0019 | 1.2085 | 0.0526 |  |
|  | Gompertz | shape | -0.0165 | -0.0288 | -0.0043 | 0.0063 | 1690.5508 | -0.0597 | -0.0803 | -0.039 | 0.0105 | 1361.4674 |
|  |  | rate | 0.0514 | 0.0413 | 0.0639 | 0.0057 |  | 0.193 | 0.1623 | 0.2296 | 0.0171 |  |
|  | Generalized Gamma | mu | 2.5731 | 2.1935 | 2.9528 | 0.1937 | 1681.251 | 0.9657 | 0.7904 | 1.141 | 0.0895 | 1286.477 |
|  |  | sigma | 1.4084 | 1.2576 | 1.5774 | 0.0814 |  | 0.9337 | 0.8413 | 1.0363 | 0.0497 |  |
|  |  | Q | -0.1351 | -0.6852 | 0.415 | 0.2807 |  | -0.9826 | -1.2682 | -0.697 | 0.1457 |  |
|  | Royston/Parmar spline model (0 knot) | gamma0 | -2.8766 | -3.2456 | -2.5076 | 0.1883 | 1694.4677 | -1.7581 | -1.9968 | -1.5193 | 0.1218 | 1395.2159 |
|  |  | gamma1 | 0.8975 | 0.7928 | 1.0023 | 0.0535 |  | 0.8733 | 0.7894 | 0.9573 | 0.0428 |  |
|  | Royston/Parmar spline model (1 knot) | gamma0 | -3.425 | -3.9579 | -2.8921 | 0.2719 | 1683.7419 | -2.5631 | -2.9084 | -2.2178 | 0.1762 | 1284.9572 |
|  |  | gamma1 | 1.4297 | 1.0851 | 1.7744 | 0.1758 |  | 2.6146 | 2.2182 | 3.011 | 0.2023 |  |
|  |  | gamma2 | 0.0489 | 0.0206 | 0.0772 | 0.0144 |  | 0.1515 | 0.1213 | 0.1817 | 0.0154 |  |
|  | Royston/Parmar spline model (2 knot) | gamma0 | -3.7001 | -4.3232 | -3.0769 | 0.3179 | 1678.4661 | -3.1179 | -3.5723 | -2.6634 | 0.2319 | 1245.5607 |
|  |  | gamma1 | 2.003 | 1.4065 | 2.5994 | 0.3043 |  | 6.5452 | 5.0923 | 7.9981 | 0.7413 |  |
|  |  | gamma2 | 0.2087 | 0.0712 | 0.3462 | 0.0701 |  | 1.4836 | 1.0446 | 1.9226 | 0.224 |  |
|  |  | gamma3 | -0.1942 | -0.3581 | -0.0303 | 0.0836 |  | -1.0483 | -1.3879 | -0.7087 | 0.1733 |  |
|  | Mixture cure model (Weibull) | theta | 0.0966 | 0.0313 | 0.2615 | NA | 1694.5131 | 0.0565 | 0.031 | 0.1007 | NA | 1374.8987 |
|  |  | shape | 0.9696 | 0.8358 | 1.1249 | 0.0735 |  | 1.0051 | 0.9105 | 1.1097 | 0.0507 |  |
|  |  | scale | 19.5698 | 14.7059 | 26.0425 | 2.8531 |  | 6.0909 | 5.2741 | 7.0343 | 0.4475 |  |
|  | Mixture cure model (Log-logistic) | theta | 0.0001 | 0 | 1 | NA | 1689.0001 | 0.049 | 0.0231 | 0.1009 | NA | 1314.2279 |
|  |  | shape | 1.2057 | 1.0755 | 1.3518 | 0.0703 |  | 1.782 | 1.5737 | 2.0179 | 0.113 |  |
|  |  | scale | 14.3804 | 12.0114 | 17.2166 | 1.3207 |  | 3.4465 | 3.0149 | 3.94 | 0.2353 |  |
|  | Mixture cure model (Exponential) | theta | 0.1087 | 0.0515 | 0.2151 | NA | 1692.685 | 0.0562 | 0.0309 | 0.1001 | NA | 1372.9092 |
|  |  | rate | 0.0525 | 0.0416 | 0.0663 | 0.0062 |  | 0.1644 | 0.1425 | 0.1896 | 0.012 |  |
|  | Mixture cure model (Log-normal) | theta | 0.0003 | 0 | 1 | NA | 1681.4877 | 0.0511 | 0.0254 | 0.1002 | NA | 1310.3968 |
|  |  | meanlog | 2.654 | 2.4765 | 2.8315 | 0.0906 |  | 1.3255 | 1.1923 | 1.4586 | 0.0679 |  |
|  |  | SDlog | 1.3885 | 1.2526 | 1.5393 | 0.073 |  | 0.9698 | 0.8724 | 1.0781 | 0.0524 |  |
|  | Mixture cure model (Gompertz) | theta | 0.0012 | 0 | 1 | NA | 1692.5571 | 0.0018 | 0 | 1 | NA | 1363.473 |
|  |  | shape | -0.0164 | -0.0264 | -0.0065 | 0.0051 |  | -0.059 | -0.0844 | -0.0335 | 0.013 |  |
|  |  | rate | 0.0515 | 0.0413 | 0.0642 | 0.0058 |  | 0.1933 | 0.1619 | 0.2308 | 0.0175 |  |
|  | Mixture cure model (Generalized Gamma) | theta | 0.0004 | 0.0000 | 1.0000 | NA | 1683.263 | 0.0089 | 0.0000 | 0.7639 | NA | 1288.444 |
|  |  | mu | 2.5700 | 2.1900 | 2.9500 | 0.1940 |  | 0.9696 | 0.7938 | 1.1455 | 0.0897 |  |
|  |  | sigma | 1.4100 | 1.2600 | 1.5800 | 0.0818 |  | 0.9237 | 0.8183 | 1.0426 | 0.0571 |  |
|  |  | Q | -0.1340 | -0.6850 | 0.4170 | 0.2810 |  | -0.9412 | -1.3131 | -0.5693 | 0.1897 |  |
|  | Non-Mixture cure model (Weibull) | theta | 0.0666 | 0.0109 | 0.3157 | NA | 1692.2888 | 0.0559 | 0.0321 | 0.0956 | NA | 1351.0327 |
|  |  | shape | 1.0444 | 0.8881 | 1.2282 | 0.0864 |  | 1.2202 | 1.1042 | 1.3483 | 0.0622 |  |
|  |  | scale | 48.7326 | 19.5292 | 121.6058 | 22.7366 |  | 12.0683 | 9.6522 | 15.089 | 1.3755 |  |
|  | Non-Mixture cure model (Log-logistic) | theta | 0.0271 | 0.0021 | 0.2683 | NA | 1691.2201 | 0.0706 | 0.0417 | 0.1172 | NA | 1325.0683 |
|  |  | shape | 1.1006 | 0.912 | 1.3283 | 0.1056 |  | 1.6405 | 1.4345 | 1.8761 | 0.1123 |  |
|  |  | scale | 54.7335 | 19.0228 | 157.4825 | 29.513 |  | 6.9873 | 5.4299 | 8.9914 | 0.899 |  |
|  | Non-Mixture cure model (Exponential) | theta | 0.0446 | 0.0068 | 0.2412 | NA | 1690.5508 | 0.0393 | 0.0165 | 0.0909 | NA | 1361.4674 |
|  |  | rate | 0.0165 | 0.0079 | 0.0347 | 0.0063 |  | 0.0597 | 0.0422 | 0.0843 | 0.0105 |  |
|  | Non-Mixture cure model (Log-normal) | theta | 0.0035 | 0 | 0.7424 | NA | 1684.1297 | 0.0597 | 0.0328 | 0.1061 | NA | 1301.346 |
|  |  | meanlog | 4.9285 | 2.7396 | 7.1174 | 1.1168 |  | 2.0432 | 1.7657 | 2.3206 | 0.1416 |  |
|  |  | SDlog | 1.9484 | 1.3992 | 2.7134 | 0.3292 |  | 1.0642 | 0.9286 | 1.2196 | 0.074 |  |
|  | Non-Mixture cure model (Gompertz) | theta | 0.0057 | 0 | 1 | NA | 1692.3448 | 0.0195 | 0.0001 | 0.8819 | NA | 1363.1732 |
|  |  | shape | -0.0084 | -0.0439 | 0.0272 | 0.0181 |  | -0.0195 | -0.1164 | 0.0773 | 0.0494 |  |
|  |  | rate | 0.0101 | 0.0003 | 0.3763 | 0.0186 |  | 0.0503 | 0.0118 | 0.2141 | 0.0372 |  |
|  | Non-Mixture cure model (Generalized Gamma) | theta | 0.0000 | 0.0000 | 1.0000 | NA | 1683.527 | 0.0073 | 0.0001 | 0.5010 | NA | 1285.58 |
|  |  | mu | 8.1600 | -1.6100 | 17.9000 | 4.9900 |  | 2.0600 | 1.3400 | 2.7700 | 0.3640 |  |
|  |  | sigma | 5.4600 | 0.6110 | 48.8000 | 6.1000 |  | 1.7300 | 0.9180 | 3.2600 | 0.5590 |  |
|  |  | Q | -1.8500 | -7.0500 | 3.3500 | 2.6500 |  | -1.7300 | -3.4900 | 0.0227 | 0.8950 |  |
| **Pembrolizumab plus Chemotherapy group, CPS≥1** | Log-logistic | shape | 1.5293 | 1.3609 | 1.7185 | 0.091 | 1724.9592 | 1.7713 | 1.5852 | 1.9792 | 0.1003 | 1571.4389 |
|  |  | scale | 17.4399 | 15.1836 | 20.0315 | 1.2328 |  | 9.4858 | 8.4084 | 10.7012 | 0.5835 |  |
|  | Weibull | shape | 1.1341 | 1.0095 | 1.274 | 0.0673 | 1727.3444 | 1.1878 | 1.0705 | 1.318 | 0.063 | 1586.1445 |
|  |  | scale | 26.0108 | 23.0367 | 29.3688 | 1.6114 |  | 14.3196 | 12.7869 | 16.0361 | 0.8271 |  |
|  | Exponential | rate | 0.0386 | 0.0336 | 0.0443 | 0.0027 | 1729.5945 | 0.0718 | 0.0629 | 0.0819 | 0.0049 | 1593.7862 |
|  | Log-normal | meanlog | 2.8256 | 2.6775 | 2.9737 | 0.0755 | 1731.9349 | 2.2299 | 2.1051 | 2.3546 | 0.0637 | 1573.7827 |
|  |  | Sdlog | 1.1696 | 1.0564 | 1.2949 | 0.0607 |  | 0.9956 | 0.9045 | 1.0958 | 0.0487 |  |
|  | Gompertz | shape | 0.0045 | -0.0073 | 0.0163 | 0.006 | 1731.0413 | 0.006 | -0.0087 | 0.0208 | 0.0075 | 1595.1601 |
|  |  | rate | 0.036 | 0.0287 | 0.0453 | 0.0042 |  | 0.0676 | 0.0553 | 0.0826 | 0.0069 |  |
|  | Generalized Gamma | mu | 3.0984 | 2.876 | 3.3209 | 0.1135 | 1726.249 | 2.3491 | 2.1452 | 2.553 | 0.104 | 1573.831 |
|  |  | sigma | 0.9991 | 0.8451 | 1.1812 | 0.0854 |  | 0.9586 | 0.8561 | 1.0733 | 0.0553 |  |
|  |  | Q | 0.601 | 0.1759 | 1.0261 | 0.2169 |  | 0.2665 | -0.1052 | 0.6382 | 0.1897 |  |
|  | Royston/Parmar spline model (0 knot) | gamma0 | -3.6953 | -4.1527 | -3.2379 | 0.2334 | 1727.3444 | -3.1615 | -3.5378 | -2.7853 | 0.192 | 1586.1445 |
|  |  | gamma1 | 1.1341 | 1.0021 | 1.266 | 0.0673 |  | 1.1878 | 1.0643 | 1.3114 | 0.063 |  |
|  | Royston/Parmar spline model (1 knot) | gamma0 | -4.1224 | -4.8637 | -3.3812 | 0.3782 | 1726.8134 | -4.0222 | -4.6846 | -3.3599 | 0.338 | 1574.0912 |
|  |  | gamma1 | 1.4468 | 1.0167 | 1.8769 | 0.2194 |  | 1.9869 | 1.5043 | 2.4695 | 0.2462 |  |
|  |  | gamma2 | 0.0348 | -0.0095 | 0.0792 | 0.0226 |  | 0.0757 | 0.0336 | 0.1179 | 0.0215 |  |
|  | Royston/Parmar spline model (2 knot) | gamma0 | -3.9797 | -4.7067 | -3.2528 | 0.3709 | 1726.8129 | -3.8136 | -4.468 | -3.1593 | 0.3339 | 1572.6517 |
|  |  | gamma1 | 1.2143 | 0.7031 | 1.7255 | 0.2608 |  | 1.5736 | 0.9508 | 2.1964 | 0.3178 |  |
|  |  | gamma2 | -0.1565 | -0.3938 | 0.0809 | 0.1211 |  | -0.1966 | -0.4492 | 0.056 | 0.1289 |  |
|  |  | gamma3 | 0.2448 | -0.0656 | 0.5551 | 0.1584 |  | 0.2998 | 0.0197 | 0.58 | 0.143 |  |
|  | Mixture cure model (Weibull) | theta | 0.1174 | 0.0571 | 0.2263 | NA | 1724.8952 | 0.0454 | 0.0151 | 0.1283 | NA | 1584.9303 |
|  |  | shape | 1.2763 | 1.1033 | 1.4764 | 0.0949 |  | 1.2662 | 1.1257 | 1.4243 | 0.076 |  |
|  |  | scale | 20.6033 | 17.1457 | 24.7581 | 1.9311 |  | 12.9735 | 11.2997 | 14.8954 | 0.9144 |  |
|  | Mixture cure model (Log-logistic) | theta | 0.0001 | 0 | 1 | NA | 1726.961 | 0.0003 | 0 | 1 | NA | 1573.4525 |
|  |  | shape | 1.5294 | 1.3609 | 1.7188 | 0.0911 |  | 1.7716 | 1.5851 | 1.9801 | 0.1006 |  |
|  |  | scale | 17.4339 | 15.1765 | 20.027 | 1.2334 |  | 9.4849 | 8.4053 | 10.7032 | 0.5848 |  |
|  | Mixture cure model (Exponential) | theta | 0.0013 | 0 | 1 | NA | 1731.6001 | 0.0002 | 0 | 1 | NA | 1595.792 |
|  |  | rate | 0.0387 | 0.0329 | 0.0455 | 0.0032 |  | 0.0718 | 0.0628 | 0.0821 | 0.0049 |  |
|  | Mixture cure model (Log-normal) | theta | 0.0004 | 0 | 1 | NA | 1733.9537 | 0.0005 | 0 | 0.9995 | NA | 1575.8158 |
|  |  | meanlog | 2.8251 | 2.6765 | 2.9737 | 0.0758 |  | 2.2292 | 2.1038 | 2.3546 | 0.064 |  |
|  |  | SDlog | 1.1693 | 1.056 | 1.2948 | 0.0608 |  | 0.995 | 0.9036 | 1.0956 | 0.0489 |  |
|  | Mixture cure model (Gompertz) | theta | 0.1436 | 0.0857 | 0.2308 | NA | 1728.7274 | 0.0123 | 0 | 0.9606 | NA | 1597.0662 |
|  |  | shape | 0.0286 | 0.0097 | 0.0475 | 0.0096 |  | 0.0095 | -0.0174 | 0.0364 | 0.0137 |  |
|  |  | rate | 0.0363 | 0.0285 | 0.0464 | 0.0045 |  | 0.0673 | 0.0549 | 0.0825 | 0.007 |  |
|  | Mixture cure model (Generalized Gamma) | theta | 0.1116 | 0.0371 | 0.2903 | NA | 1726.866 | 0.0002 | 0.0000 | 1.0000 | NA | 1575.834 |
|  |  | mu | 3.0182 | 2.8147 | 3.2217 | 0.1038 |  | 2.3500 | 2.1400 | 2.5500 | 0.1040 |  |
|  |  | sigma | 0.8025 | 0.5764 | 1.1174 | 0.1355 |  | 0.9580 | 0.8540 | 1.0800 | 0.0564 |  |
|  |  | Q | 0.9457 | 0.3123 | 1.5791 | 0.3232 |  | 0.2670 | -0.1070 | 0.6410 | 0.1910 |  |
|  | Non-Mixture cure model (Weibull) | theta | 0.0691 | 0.0128 | 0.2976 | NA | 1724.9296 | 0.0379 | 0.011 | 0.1222 | NA | 1576.8469 |
|  |  | shape | 1.345 | 1.1358 | 1.5926 | 0.116 |  | 1.4524 | 1.2766 | 1.6525 | 0.0956 |  |
|  |  | scale | 43.4123 | 21.9659 | 85.7981 | 15.0895 |  | 26.4456 | 17.9383 | 38.9874 | 5.2373 |  |
|  | Non-Mixture cure model (Log-logistic) | theta | 0.013 | 0.0004 | 0.3234 | NA | 1725.1113 | 0.0184 | 0.0032 | 0.0984 | NA | 1574.1162 |
|  |  | shape | 1.3582 | 1.1217 | 1.6445 | 0.1326 |  | 1.5823 | 1.349 | 1.856 | 0.1288 |  |
|  |  | scale | 60.3257 | 23.1859 | 156.9567 | 29.4312 |  | 25.8481 | 15.9014 | 42.0165 | 6.4071 |  |
|  | Non-Mixture cure model (Exponential) | theta | 0 | 0 | 1 | NA | 1731.8471 | 0 | 0 | 1 | NA | 1596.0956 |
|  |  | rate | 0.0009 | 0.0002 | 0.0048 | 0.0008 |  | 0.0014 | 0.0001 | 0.0357 | 0.0023 |  |
|  | Non-Mixture cure model (Log-normal) | theta | 0 | 0 | 1 | NA | 1726.0591 | 0 | 0 | 0.9689 | NA | 1573.2845 |
|  |  | meanlog | 7.526 | 1.5767 | 13.4752 | 3.0354 |  | 4.5958 | 2.7035 | 6.4881 | 0.9655 |  |
|  |  | SDlog | 2.241 | 1.3245 | 3.7917 | 0.6013 |  | 1.5792 | 1.1412 | 2.1854 | 0.2617 |  |
|  | Non-Mixture cure model (Gompertz) | theta | 0.1426 | 0.0847 | 0.2303 | NA | 1728.0622 | 0.0293 | 0.0053 | 0.1471 | NA | 1595.1873 |
|  |  | shape | 0.0459 | 0.0255 | 0.0662 | 0.0104 |  | 0.0403 | 0.0132 | 0.0674 | 0.0138 |  |
|  |  | rate | 0.0153 | 0.0112 | 0.0209 | 0.0024 |  | 0.0177 | 0.011 | 0.0285 | 0.0043 |  |
|  | Non-Mixture cure model (Generalized Gamma) | theta | 0.1039 | 0.0197 | 0.4005 | NA | 1726.765 | 0.0015 | 0.0000 | 1.0000 | NA | 1575.238 |
|  |  | mu | 3.6180 | 2.9783 | 4.2577 | 0.3264 |  | 4.0200 | 1.0800 | 6.9600 | 1.5000 |  |
|  |  | sigma | 0.6285 | 0.3021 | 1.3076 | 0.2349 |  | 1.2800 | 0.3610 | 4.5700 | 0.8320 |  |
|  |  | Q | 1.2328 | 0.1401 | 2.3255 | 0.5575 |  | 0.1960 | -1.1400 | 1.5400 | 0.6840 |  |
| **Chemotherapy group, CPS≥10** | Log-logistic | shape | 1.724 | 1.425 | 2.0858 | 0.1676 | 615.9074 | 2.1568 | 1.7933 | 2.5939 | 0.2031 | 534.5474 |
|  |  | scale | 15.6818 | 12.747 | 19.2923 | 1.6578 |  | 8.456 | 7.1549 | 9.9936 | 0.7208 |  |
|  | Weibull | shape | 1.207 | 1.0016 | 1.4544 | 0.1148 | 620.4188 | 1.3799 | 1.1689 | 1.629 | 0.1168 | 540.0631 |
|  |  | scale | 23.3315 | 19.3189 | 28.1776 | 2.2466 |  | 11.9865 | 10.1809 | 14.1122 | 0.9984 |  |
|  | Exponential | rate | 0.0436 | 0.0347 | 0.0546 | 0.005 | 622.0071 | 0.0884 | 0.071 | 0.11 | 0.0099 | 550.2208 |
|  | Log-normal | meanlog | 2.7351 | 2.5171 | 2.9532 | 0.1113 | 618.1349 | 2.1058 | 1.9294 | 2.2822 | 0.09 | 536.6672 |
|  |  | Sdlog | 1.0314 | 0.8733 | 1.2182 | 0.0876 |  | 0.8354 | 0.7144 | 0.977 | 0.0667 |  |
|  | Gompertz | shape | 0.0064 | -0.0132 | 0.0259 | 0.01 | 623.6054 | 0.0264 | -0.0003 | 0.053 | 0.0136 | 548.7852 |
|  |  | rate | 0.0397 | 0.0274 | 0.0575 | 0.0075 |  | 0.0705 | 0.0505 | 0.0983 | 0.012 |  |
|  | Generalized Gamma | mu | 2.893 | 2.548 | 3.237 | 0.176 | 618.9743 | 2.2466 | 1.9773 | 2.516 | 0.1374 | 537.0379 |
|  |  | sigma | 0.963 | 0.773 | 1.199 | 0.108 |  | 0.7939 | 0.664 | 0.9492 | 0.0724 |  |
|  |  | Q | 0.36 | -0.291 | 1.011 | 0.332 |  | 0.3629 | -0.1853 | 0.9112 | 0.2797 |  |
|  | Royston/Parmar spline model (0 knot) | gamma0 | -3.8017 | -4.5705 | -3.0329 | 0.3923 | 620.4188 | -3.4273 | -4.0858 | -2.7687 | 0.336 | 540.0631 |
|  |  | gamma1 | 1.207 | 0.9819 | 1.432 | 0.1148 |  | 1.3799 | 1.1509 | 1.6089 | 0.1168 |  |
|  | Royston/Parmar spline model (1 knot) | gamma0 | -4.857 | -6.3514 | -3.3627 | 0.7624 | 618.86 | -4.4752 | -5.7407 | -3.2097 | 0.6457 | 537.0171 |
|  |  | gamma1 | 1.8502 | 1.0745 | 2.626 | 0.3958 |  | 2.1692 | 1.3677 | 2.9707 | 0.4089 |  |
|  |  | gamma2 | 0.0973 | -0.0094 | 0.204 | 0.0544 |  | 0.1182 | 0.0089 | 0.2275 | 0.0558 |  |
|  | Royston/Parmar spline model (2 knot) | gamma0 | -4.3814 | -5.8885 | -2.8743 | 0.7689 | 619.361 | -3.9972 | -5.2201 | -2.7743 | 0.6239 | 536.1503 |
|  |  | gamma1 | 1.4157 | 0.4787 | 2.3526 | 0.478 |  | 1.5617 | 0.6159 | 2.5075 | 0.4826 |  |
|  |  | gamma2 | -0.2933 | -0.815 | 0.2285 | 0.2662 |  | -0.5861 | -1.3118 | 0.1395 | 0.3702 |  |
|  |  | gamma3 | 0.4523 | -0.1647 | 1.0694 | 0.3148 |  | 0.7266 | -0.0293 | 1.4824 | 0.3857 |  |
|  | Mixture cure model (Weibull) | theta | 0.1204 | 0.0539 | 0.2473 | NA | 618.4182 | 0.0267 | 0.0044 | 0.1455 | NA | 540.1834 |
|  |  | shape | 1.4257 | 1.1504 | 1.767 | 0.1561 |  | 1.4724 | 1.2325 | 1.759 | 0.1336 |  |
|  |  | scale | 18.252 | 14.7798 | 22.5399 | 1.965 |  | 11.2812 | 9.5437 | 13.3349 | 0.9627 |  |
|  | Mixture cure model (Log-logistic) | theta | 0.0124 | 0 | 0.999 | NA | 617.8975 | 0.0001 | 0 | 1 | NA | 536.5528 |
|  |  | shape | 1.7507 | 1.3541 | 2.2634 | 0.2294 |  | 2.1572 | 1.7934 | 2.5949 | 0.2033 |  |
|  |  | scale | 15.3947 | 11.5437 | 20.5303 | 2.2612 |  | 8.4548 | 7.1535 | 9.9929 | 0.721 |  |
|  | Mixture cure model (Exponential) | theta | 0.013 | 0 | 0.9796 | NA | 624.0535 | 0.0001 | 0 | 1 | NA | 552.2241 |
|  |  | rate | 0.0449 | 0.0325 | 0.062 | 0.0074 |  | 0.0884 | 0.071 | 0.11 | 0.0099 |  |
|  | Mixture cure model (Log-normal) | theta | 0.0004 | 0 | 1 | NA | 620.1385 | 0.0005 | 0 | 1 | NA | 538.6864 |
|  |  | meanlog | 2.7345 | 2.5141 | 2.9549 | 0.1125 |  | 2.105 | 1.928 | 2.282 | 0.0903 |  |
|  |  | SDlog | 1.0311 | 0.872 | 1.2193 | 0.0882 |  | 0.8348 | 0.7133 | 0.977 | 0.067 |  |
|  | Mixture cure model (Gompertz) | theta | 0.1294 | 0.0636 | 0.2453 | NA | 622.6051 | 0.0231 | 0.0029 | 0.1596 | NA | 549.7399 |
|  |  | shape | 0.0356 | 0.0079 | 0.0633 | 0.0141 |  | 0.0396 | 0.0072 | 0.0719 | 0.0165 |  |
|  |  | rate | 0.0379 | 0.0255 | 0.0565 | 0.0077 |  | 0.0681 | 0.0484 | 0.0959 | 0.0119 |  |
|  | Mixture cure model (Generalized Gamma) | theta | 0.1021 | 0.0269 | 0.3184 | NA | 619.9762 | 0.0110 | 0.0000 | 0.8057 | NA | 538.9349 |
|  |  | mu | 2.8440 | 2.5529 | 3.1351 | 0.1485 |  | 2.2516 | 1.9885 | 2.5148 | 0.1343 |  |
|  |  | sigma | 0.7737 | 0.5234 | 1.1437 | 0.1543 |  | 0.7681 | 0.5933 | 0.9946 | 0.1013 |  |
|  |  | Q | 0.7267 | -0.0934 | 1.5469 | 0.4184 |  | 0.4321 | -0.2306 | 1.0949 | 0.3382 |  |
|  | Non-Mixture cure model (Weibull) | theta | 0.0984 | 0.0286 | 0.2879 | NA | 617.7959 | 0.0216 | 0.0025 | 0.1615 | NA | 536.775 |
|  |  | shape | 1.5618 | 1.2308 | 1.9817 | 0.1898 |  | 1.7108 | 1.3991 | 2.092 | 0.1756 |  |
|  |  | scale | 30.6732 | 18.034 | 52.1706 | 8.3121 |  | 22.4203 | 13.8124 | 36.3925 | 5.5411 |  |
|  | Non-Mixture cure model (Log-logistic) | theta | 0.0416 | 0.0042 | 0.3078 | NA | 617.6936 | 0.0089 | 0.0004 | 0.1884 | NA | 535.9002 |
|  |  | shape | 1.642 | 1.232 | 2.1886 | 0.2407 |  | 1.8539 | 1.4425 | 2.3826 | 0.2373 |  |
|  |  | scale | 34.1025 | 15.5707 | 74.6901 | 13.6409 |  | 22.2186 | 11.6204 | 42.4828 | 7.3478 |  |
|  | Non-Mixture cure model (Exponential) | theta | 0 | 0 | 1 | NA | 624.2478 | 0 | 0 | 1 | NA | 552.88 |
|  |  | rate | 0.0017 | 0 | 0.5758 | 0.0051 |  | 0.0025 | 0.0004 | 0.0182 | 0.0025 |  |
|  | Non-Mixture cure model (Log-normal) | theta | 0.0001 | 0 | 1 | NA | 618.6346 | 0 | 0 | 1 | NA | 536.5535 |
|  |  | meanlog | 5.0703 | 1.2482 | 8.8924 | 1.9501 |  | 4.354 | 1.7993 | 6.9088 | 1.3035 |  |
|  |  | SDlog | 1.5964 | 0.85 | 2.9979 | 0.5133 |  | 1.3865 | 0.8488 | 2.2646 | 0.3471 |  |
|  | Non-Mixture cure model (Gompertz) | theta | 0.1261 | 0.0612 | 0.2421 | NA | 622.0043 | 0.0207 | 0.0035 | 0.1129 | NA | 547.2242 |
|  |  | shape | 0.056 | 0.0262 | 0.0858 | 0.0152 |  | 0.0793 | 0.042 | 0.1166 | 0.019 |  |
|  |  | rate | 0.015 | 0.0092 | 0.0244 | 0.0037 |  | 0.0152 | 0.0087 | 0.0267 | 0.0044 |  |
|  | Non-Mixture cure model (Generalized Gamma) | theta | 0.0878 | 0.0101 | 0.4753 | NA | 619.7633 | 0.0093 | 0.0000 | 0.8250 | NA | 538.1314 |
|  |  | mu | 3.4577 | 2.6764 | 4.2391 | 0.3986 |  | 3.2600 | 2.0200 | 4.5100 | 0.6350 |  |
|  |  | sigma | 0.6880 | 0.2913 | 1.6249 | 0.3017 |  | 0.7720 | 0.2930 | 2.0300 | 0.3810 |  |
|  |  | Q | 0.8927 | -0.2861 | 2.0715 | 0.6014 |  | 0.6060 | -0.5310 | 1.7400 | 0.5800 |  |
| **Pembrolizumab group, CPS≥10** | Log-logistic | shape | 0.926 | 0.7496 | 1.144 | 0.0999 | 551.5416 | 1.2238 | 1.0166 | 1.4733 | 0.1158 | 493.6351 |
|  |  | scale | 20.6013 | 13.8607 | 30.6199 | 4.1655 |  | 5.1841 | 3.8519 | 6.977 | 0.7856 |  |
|  | Weibull | shape | 0.7427 | 0.5954 | 0.9264 | 0.0838 | 552.5481 | 0.769 | 0.6444 | 0.9176 | 0.0693 | 510.9634 |
|  |  | scale | 36.8856 | 26.1979 | 51.9334 | 6.4389 |  | 10.6602 | 7.9219 | 14.345 | 1.6147 |  |
|  | Exponential | rate | 0.0284 | 0.0221 | 0.0365 | 0.0036 | 558.3681 | 0.0868 | 0.0692 | 0.1088 | 0.01 | 518.6736 |
|  | Log-normal | meanlog | 3.0061 | 2.599 | 3.4131 | 0.2077 | 549.7261 | 1.7265 | 1.4364 | 2.0166 | 0.148 | 491.7227 |
|  |  | Sdlog | 1.8325 | 1.5142 | 2.2177 | 0.1784 |  | 1.3779 | 1.1669 | 1.6271 | 0.1169 |  |
|  | Gompertz | shape | -0.0282 | -0.0503 | -0.0061 | 0.0113 | 553.557 | -0.0743 | -0.1088 | -0.0398 | 0.0176 | 495.5539 |
|  |  | rate | 0.0445 | 0.0299 | 0.0663 | 0.009 |  | 0.1608 | 0.1182 | 0.2188 | 0.0253 |  |
|  | Generalized Gamma | mu | 3.0077 | 2.1612 | 3.8542 | 0.4319 | 551.7261 | 1.164 | 0.702 | 1.626 | 0.236 | 486.7895 |
|  |  | sigma | 1.8316 | 1.3864 | 2.4198 | 0.2602 |  | 1.281 | 1.069 | 1.535 | 0.118 |  |
|  |  | Q | 0.0023 | -1.0608 | 1.0654 | 0.5424 |  | -0.898 | -1.513 | -0.283 | 0.314 |  |
|  | Royston/Parmar spline model (0 knot) | gamma0 | -2.6794 | -3.2862 | -2.0727 | 0.3096 | 552.5481 | -1.8198 | -2.238 | -1.4016 | 0.2134 | 510.9634 |
|  |  | gamma1 | 0.7427 | 0.5785 | 0.9068 | 0.0838 |  | 0.769 | 0.6331 | 0.9049 | 0.0693 |  |
|  | Royston/Parmar spline model (1 knot) | gamma0 | -2.9064 | -3.6239 | -2.1889 | 0.3661 | 552.0454 | -2.3289 | -2.8726 | -1.7853 | 0.2774 | 485.4695 |
|  |  | gamma1 | 1.0842 | 0.5871 | 1.5812 | 0.2536 |  | 2.1191 | 1.4853 | 2.7529 | 0.3234 |  |
|  |  | gamma2 | 0.0263 | -0.0077 | 0.0603 | 0.0173 |  | 0.1039 | 0.0607 | 0.1471 | 0.022 |  |
|  | Royston/Parmar spline model (2 knot) | gamma0 | -2.9055 | -3.6444 | -2.1666 | 0.377 | 550.4197 | -2.2851 | -2.8311 | -1.739 | 0.2786 | 484.4843 |
|  |  | gamma1 | 1.6391 | 0.7781 | 2.5001 | 0.4393 |  | 3.2239 | 1.7631 | 4.6846 | 0.7453 |  |
|  |  | gamma2 | 0.1455 | 0.0059 | 0.2851 | 0.0712 |  | 0.3712 | 0.052 | 0.6905 | 0.1629 |  |
|  |  | gamma3 | -0.1711 | -0.3657 | 0.0235 | 0.0993 |  | -0.2247 | -0.4944 | 0.045 | 0.1376 |  |
|  | Mixture cure model (Weibull) | theta | 0.1232 | 0.0042 | 0.8227 | NA | 554.314 | 0.1311 | 0.0711 | 0.2293 | NA | 499.4404 |
|  |  | shape | 0.7875 | 0.5788 | 1.0716 | 0.1238 |  | 0.9964 | 0.8306 | 1.1952 | 0.0925 |  |
|  |  | scale | 26.9932 | 9.3072 | 78.2875 | 14.6648 |  | 6.6074 | 5.114 | 8.5369 | 0.8637 |  |
|  | Mixture cure model (Log-logistic) | theta | 0.0011 | 0 | 1 | NA | 553.5447 | 0.1044 | 0.044 | 0.228 | NA | 490.2351 |
|  |  | shape | 0.9266 | 0.7482 | 1.1475 | 0.1011 |  | 1.5013 | 1.1987 | 1.8802 | 0.1724 |  |
|  |  | scale | 20.5492 | 13.5633 | 31.1333 | 4.3558 |  | 4.0247 | 3.0145 | 5.3735 | 0.5935 |  |
|  | Mixture cure model (Exponential) | theta | 0.2467 | 0.1333 | 0.411 | NA | 555.219 | 0.131 | 0.0713 | 0.2283 | NA | 497.4416 |
|  |  | rate | 0.0516 | 0.0335 | 0.0794 | 0.0114 |  | 0.1511 | 0.1173 | 0.1946 | 0.0195 |  |
|  | Mixture cure model (Log-normal) | theta | 0.0006 | 0 | 1 | NA | 551.729 | 0.1118 | 0.0506 | 0.229 | NA | 487.8845 |
|  |  | meanlog | 3.0046 | 2.5909 | 3.4184 | 0.2111 |  | 1.4186 | 1.1342 | 1.7031 | 0.1451 |  |
|  |  | SDlog | 1.8318 | 1.5122 | 2.219 | 0.1792 |  | 1.1158 | 0.9143 | 1.3617 | 0.1134 |  |
|  | Mixture cure model (Gompertz) | theta | 0.0214 | 0 | 1 | NA | 555.5578 | 0.0172 | 0 | 1 | NA | 497.5558 |
|  |  | shape | -0.0273 | -0.0594 | 0.0048 | 0.0164 |  | -0.0706 | -0.2126 | 0.0714 | 0.0724 |  |
|  |  | rate | 0.0456 | 0.0211 | 0.0983 | 0.0179 |  | 0.1635 | 0.0851 | 0.3141 | 0.0545 |  |
|  | Mixture cure model (Generalized Gamma) | theta | 0.0011 | 0.0000 | 1.0000 | NA | 553.7317 | 0.0703 | 0.0075 | 0.4323 | NA | 488.2904 |
|  |  | mu | 3.0100 | 2.1300 | 3.8800 | 0.4460 |  | 1.2020 | 0.7535 | 1.6506 | 0.2289 |  |
|  |  | sigma | 1.8300 | 1.3700 | 2.4400 | 0.2680 |  | 1.1804 | 0.9124 | 1.5271 | 0.1551 |  |
|  |  | Q | 0.0041 | -1.1000 | 1.1100 | 0.5660 |  | -0.5781 | -1.5543 | 0.3980 | 0.4980 |  |
|  | Non-Mixture cure model (Weibull) | theta | 0.0763 | 0.0003 | 0.9541 | NA | 554.0031 | 0.1267 | 0.0675 | 0.2253 | NA | 495.5806 |
|  |  | shape | 0.8282 | 0.5956 | 1.1515 | 0.1393 |  | 1.1515 | 0.9579 | 1.3842 | 0.1081 |  |
|  |  | scale | 86.5829 | 3.1981 | 2344.042 | 145.715 |  | 11.4245 | 7.9159 | 16.4883 | 2.1386 |  |
|  | Non-Mixture cure model (Log-logistic) | theta | 0.0271 | 0 | 0.9798 | NA | 553.8616 | 0.1145 | 0.0534 | 0.2286 | NA | 491.6717 |
|  |  | shape | 0.8572 | 0.5902 | 1.2451 | 0.1633 |  | 1.4121 | 1.1147 | 1.7889 | 0.1704 |  |
|  |  | scale | 112.834 | 3.1744 | 4010.6764 | 205.5688 |  | 8.1548 | 4.9502 | 13.434 | 2.0769 |  |
|  | Non-Mixture cure model (Exponential) | theta | 0.2059 | 0.0816 | 0.4306 | NA | 553.557 | 0.1148 | 0.0533 | 0.2301 | NA | 495.5539 |
|  |  | rate | 0.0282 | 0.0129 | 0.0618 | 0.0113 |  | 0.0743 | 0.0467 | 0.1182 | 0.0176 |  |
|  | Non-Mixture cure model (Log-normal) | theta | 0.0012 | 0 | 1 | NA | 552.2092 | 0.1035 | 0.0413 | 0.2365 | NA | 487.2032 |
|  |  | meanlog | 6.3885 | -1.1489 | 13.9259 | 3.8457 |  | 2.1886 | 1.5659 | 2.8113 | 0.3177 |  |
|  |  | SDlog | 2.6574 | 1.2711 | 5.5556 | 0.9999 |  | 1.2539 | 0.9684 | 1.6235 | 0.1653 |  |
|  | Non-Mixture cure model (Gompertz) | theta | 0.0963 | 0.0004 | 0.9645 | NA | 555.102 | 0.1178 | 0.0454 | 0.2724 | NA | 497.5482 |
|  |  | shape | -0.0149 | -0.0591 | 0.0292 | 0.0225 |  | 0.0034 | -0.0801 | 0.0868 | 0.0426 |  |
|  |  | rate | 0.0201 | 0.0021 | 0.1886 | 0.0229 |  | 0.0745 | 0.0476 | 0.1167 | 0.0171 |  |
|  | Non-Mixture cure model (Generalized Gamma) | theta | 0.0000 | 0.0000 | 1.0000 | NA | 553.7751 | 0.0392 | 0.0003 | 0.8695 | NA | 487.9773 |
|  |  | mu | 9.1300 | -3.9900 | 22.3000 | 6.7000 |  | 2.3554 | 0.8696 | 3.8412 | 0.7581 |  |
|  |  | sigma | 5.3100 | 0.4230 | 66.6000 | 6.8500 |  | 1.7997 | 0.5828 | 5.5574 | 1.0353 |  |
|  |  | Q | -1.0800 | -6.1700 | 4.0000 | 2.5900 |  | -0.9282 | -3.5475 | 1.6912 | 1.3365 |  |
| **Pembrolizumab plus Chemotherapy group, CPS≥10** | Log-logistic | shape | 1.3949 | 1.1463 | 1.6974 | 0.1397 | 632.2635 | 1.5138 | 1.2601 | 1.8185 | 0.1417 | 595.7447 |
|  |  | scale | 17.9938 | 14.0701 | 23.0118 | 2.2583 |  | 9.6529 | 7.6825 | 12.1286 | 1.1244 |  |
|  | Weibull | shape | 1.0375 | 0.8527 | 1.2623 | 0.1038 | 635.6249 | 1.0328 | 0.8664 | 1.2311 | 0.0925 | 603.5464 |
|  |  | scale | 27.809 | 22.2893 | 34.6956 | 3.1392 |  | 15.6754 | 12.662 | 19.406 | 1.7074 |  |
|  | Exponential | rate | 0.0359 | 0.0285 | 0.0452 | 0.0042 | 633.7579 | 0.0641 | 0.0515 | 0.0798 | 0.0072 | 601.6739 |
|  | Log-normal | meanlog | 2.8747 | 2.6074 | 3.1421 | 0.1364 | 635.1655 | 2.2674 | 2.0349 | 2.5 | 0.1187 | 595.0875 |
|  |  | Sdlog | 1.2894 | 1.0858 | 1.5312 | 0.113 |  | 1.1422 | 0.9728 | 1.3413 | 0.0936 |  |
|  | Gompertz | shape | -0.0072 | -0.0276 | 0.0131 | 0.0104 | 635.261 | -0.0124 | -0.037 | 0.0122 | 0.0125 | 602.6555 |
|  |  | rate | 0.0399 | 0.0276 | 0.0577 | 0.0075 |  | 0.0728 | 0.0525 | 0.101 | 0.0122 |  |
|  | Generalized Gamma | mu | 3.098 | 2.699 | 3.497 | 0.204 | 635.5129 | 2.2353 | 1.8027 | 2.6678 | 0.2207 | 597.0572 |
|  |  | sigma | 1.141 | 0.868 | 1.499 | 0.159 |  | 1.1492 | 0.9667 | 1.3663 | 0.1014 |  |
|  |  | Q | 0.465 | -0.232 | 1.161 | 0.355 |  | -0.0632 | -0.7759 | 0.6495 | 0.3636 |  |
|  | Royston/Parmar spline model (0 knot) | gamma0 | -3.45 | -4.1567 | -2.7432 | 0.3606 | 635.6249 | -2.8423 | -3.4099 | -2.2747 | 0.2896 | 603.5464 |
|  |  | gamma1 | 1.0375 | 0.834 | 1.241 | 0.1038 |  | 1.0328 | 0.8514 | 1.2142 | 0.0925 |  |
|  | Royston/Parmar spline model (1 knot) | gamma0 | -3.9937 | -5.1498 | -2.8375 | 0.5899 | 635.8001 | -3.7262 | -4.6753 | -2.7772 | 0.4842 | 597.0577 |
|  |  | gamma1 | 1.4711 | 0.7557 | 2.1866 | 0.365 |  | 1.8282 | 1.1898 | 2.4665 | 0.3257 |  |
|  |  | gamma2 | 0.0488 | -0.0255 | 0.1231 | 0.0379 |  | 0.0884 | 0.0252 | 0.1516 | 0.0323 |  |
|  | Royston/Parmar spline model (2 knot) | gamma0 | -3.7986 | -4.8336 | -2.7637 | 0.528 | 633.8657 | -3.5198 | -4.4528 | -2.5867 | 0.4761 | 597.4788 |
|  |  | gamma1 | 0.9722 | 0.2443 | 1.7001 | 0.3714 |  | 1.4582 | 0.652 | 2.2644 | 0.4113 |  |
|  |  | gamma2 | -0.3645 | -0.7346 | 0.0055 | 0.1888 |  | -0.2161 | -0.6132 | 0.1811 | 0.2026 |  |
|  |  | gamma3 | 0.5279 | 0.0408 | 1.0149 | 0.2485 |  | 0.3135 | -0.0973 | 0.7244 | 0.2096 |  |
|  | Mixture cure model (Weibull) | theta | 0.1978 | 0.1049 | 0.3416 | NA | 633.3559 | 0.0787 | 0.0181 | 0.2841 | NA | 604.1184 |
|  |  | shape | 1.2735 | 1.0091 | 1.6073 | 0.1512 |  | 1.1186 | 0.9143 | 1.3687 | 0.1151 |  |
|  |  | scale | 18.0839 | 13.7038 | 23.864 | 2.559 |  | 13.1087 | 9.7131 | 17.6913 | 2.0051 |  |
|  | Mixture cure model (Log-logistic) | theta | 0.0428 | 0.0002 | 0.9143 | NA | 634.1431 | 0.0098 | 0 | 1 | NA | 597.7154 |
|  |  | shape | 1.4541 | 1.078 | 1.9615 | 0.2221 |  | 1.5326 | 1.1361 | 2.0674 | 0.2341 |  |
|  |  | scale | 16.7242 | 10.6795 | 26.1902 | 3.8273 |  | 9.4894 | 6.4002 | 14.0699 | 1.9069 |  |
|  | Mixture cure model (Exponential) | theta | 0.1077 | 0.0204 | 0.4121 | NA | 634.6555 | 0.044 | 0.0028 | 0.4346 | NA | 603.1861 |
|  |  | rate | 0.0454 | 0.0291 | 0.0708 | 0.0103 |  | 0.0713 | 0.05 | 0.1018 | 0.0129 |  |
|  | Mixture cure model (Log-normal) | theta | 0.0006 | 0 | 1 | NA | 637.1697 | 0.0009 | 0 | 1 | NA | 597.0909 |
|  |  | meanlog | 2.8736 | 2.6022 | 3.1451 | 0.1385 |  | 2.2658 | 2.0212 | 2.5104 | 0.1248 |  |
|  |  | SDlog | 1.2889 | 1.0842 | 1.5322 | 0.1137 |  | 1.1413 | 0.9665 | 1.3477 | 0.0968 |  |
|  | Mixture cure model (Gompertz) | theta | 0.2086 | 0.1166 | 0.3447 | NA | 635.0704 | 0.0003 | 0 | 1 | NA | 604.6566 |
|  |  | shape | 0.0261 | -0.005 | 0.0572 | 0.0159 |  | -0.0123 | -0.031 | 0.0063 | 0.0095 |  |
|  |  | rate | 0.0429 | 0.029 | 0.0633 | 0.0085 |  | 0.0729 | 0.0525 | 0.1012 | 0.0122 |  |
|  | Mixture cure model (Generalized Gamma) | theta | 0.1992 | 0.0992 | 0.3600 | NA | 635.3532 | 0.0003 | 0.0000 | 1.0000 | NA | 599.0595 |
|  |  | mu | 2.8990 | 2.5878 | 3.2102 | 0.1588 |  | 2.2400 | 1.8000 | 2.6700 | 0.2210 |  |
|  |  | sigma | 0.7787 | 0.5270 | 1.1507 | 0.1551 |  | 1.1500 | 0.9640 | 1.3700 | 0.1030 |  |
|  |  | Q | 1.0205 | 0.2438 | 1.7973 | 0.3963 |  | -0.0622 | -0.7770 | 0.6530 | 0.3650 |  |
|  | Non-Mixture cure model (Weibull) | theta | 0.1693 | 0.0637 | 0.3792 | NA | 633.3781 | 0.0733 | 0.0174 | 0.2612 | NA | 600.5118 |
|  |  | shape | 1.364 | 1.0556 | 1.7625 | 0.1784 |  | 1.2899 | 1.0453 | 1.5918 | 0.1384 |  |
|  |  | scale | 29.4789 | 15.8014 | 54.9954 | 9.3789 |  | 24.865 | 13.1164 | 47.1372 | 8.1143 |  |
|  | Non-Mixture cure model (Log-logistic) | theta | 0.0751 | 0.0096 | 0.4038 | NA | 633.6643 | 0.0553 | 0.0103 | 0.2487 | NA | 598.41 |
|  |  | shape | 1.4044 | 1.0317 | 1.9118 | 0.221 |  | 1.481 | 1.1326 | 1.9367 | 0.2027 |  |
|  |  | scale | 36.1512 | 13.5154 | 96.6979 | 18.1475 |  | 20.8759 | 10.0627 | 43.3088 | 7.7728 |  |
|  | Non-Mixture cure model (Exponential) | theta | 0.0041 | 0 | 0.9998 | NA | 635.261 | 0.0028 | 0 | 0.9887 | NA | 602.6555 |
|  |  | rate | 0.0073 | 0.0005 | 0.1169 | 0.0103 |  | 0.0124 | 0.0017 | 0.0897 | 0.0125 |  |
|  | Non-Mixture cure model (Log-normal) | theta | 0 | 0 | 1 | NA | 635.2862 | 0.0099 | 0 | 0.7222 | NA | 597.166 |
|  |  | meanlog | 6.2235 | 0.1983 | 12.2486 | 3.0741 |  | 3.8005 | 1.9712 | 5.6298 | 0.9333 |  |
|  |  | SDlog | 2.0803 | 1.0475 | 4.1315 | 0.7282 |  | 1.4891 | 0.9864 | 2.2481 | 0.313 |  |
|  | Non-Mixture cure model (Gompertz) | theta | 0.2065 | 0.1169 | 0.3383 | NA | 634.8806 | 0.0076 | 0 | 1 | NA | 604.6555 |
|  |  | shape | 0.0435 | 0.0116 | 0.0755 | 0.0163 |  | 0.0031 | -0.1124 | 0.1187 | 0.059 |  |
|  |  | rate | 0.0208 | 0.013 | 0.0333 | 0.005 |  | 0.0149 | 0 | 5.6655 | 0.0452 |  |
|  | Non-Mixture cure model (Generalized Gamma) | theta | 0.1904 | 0.0813 | 0.3845 | NA | 635.2203 | 0.0000 | 0.0000 | 1.0000 | NA | 598.9603 |
|  |  | mu | 3.3373 | 2.8528 | 3.8218 | 0.2472 |  | 5.5700 | -10.0000 | 21.2000 | 7.9600 |  |
|  |  | sigma | 0.6497 | 0.3623 | 1.1653 | 0.1937 |  | 2.9800 | 0.0227 | 393.0000 | 7.4300 |  |
|  |  | Q | 1.1936 | 0.2503 | 2.1369 | 0.4813 |  | -0.9970 | -9.0700 | 7.0800 | 4.1200 |  |
| **Abbreviations: *CPS*** combined positive score, ***AIC*** Akaike information criterion, ***Est*** point estimation, ***L95%*** lower boundary of 95% confidence interval, ***U95%*** upper boundary of 95% confidence interval, ***NA*** not available, ***OS*** overall survival, ***PFS*** progression-free survival. | | | | | | | | | | | | |

Supplemental Figure 1. Diagram of modeled PFS and OS fit curves in different regimens. Supplemental Figure 1A: Output in the CPS≥1 group; Supplemental Figure 1B: Output in the CPS≥10 group. The colored lines represent the modeled survival curves, the black lines represent the Kaplan-Meier survival curves. Each cycle of the x-axis is 3 weeks. PFS progression-free survival; OS overall survival; CPS combined positive score; C chemotherapy group; P pembrolizumab group; PC pembrolizumab plus chemotherapy group.

Supplemental Figure 2. Tornado diagram of one-way sensitivity analysis. (A)The output of PC vs C in the Chinese setting. (B) The output of P vs C in the Chinese setting. ***QALY*** quality-adjusted life-year, ***PD*** progressed disease, ***PFS*** progression-free survival, ***BSC*** best supportive care, ***BSA*** body surface area, ***ICUR*** Incremental cost-utility ratio, ***C*** chemotherapy, ***PC*** pembrolizumab plus chemotherapy, ***AEs*** adverse events.


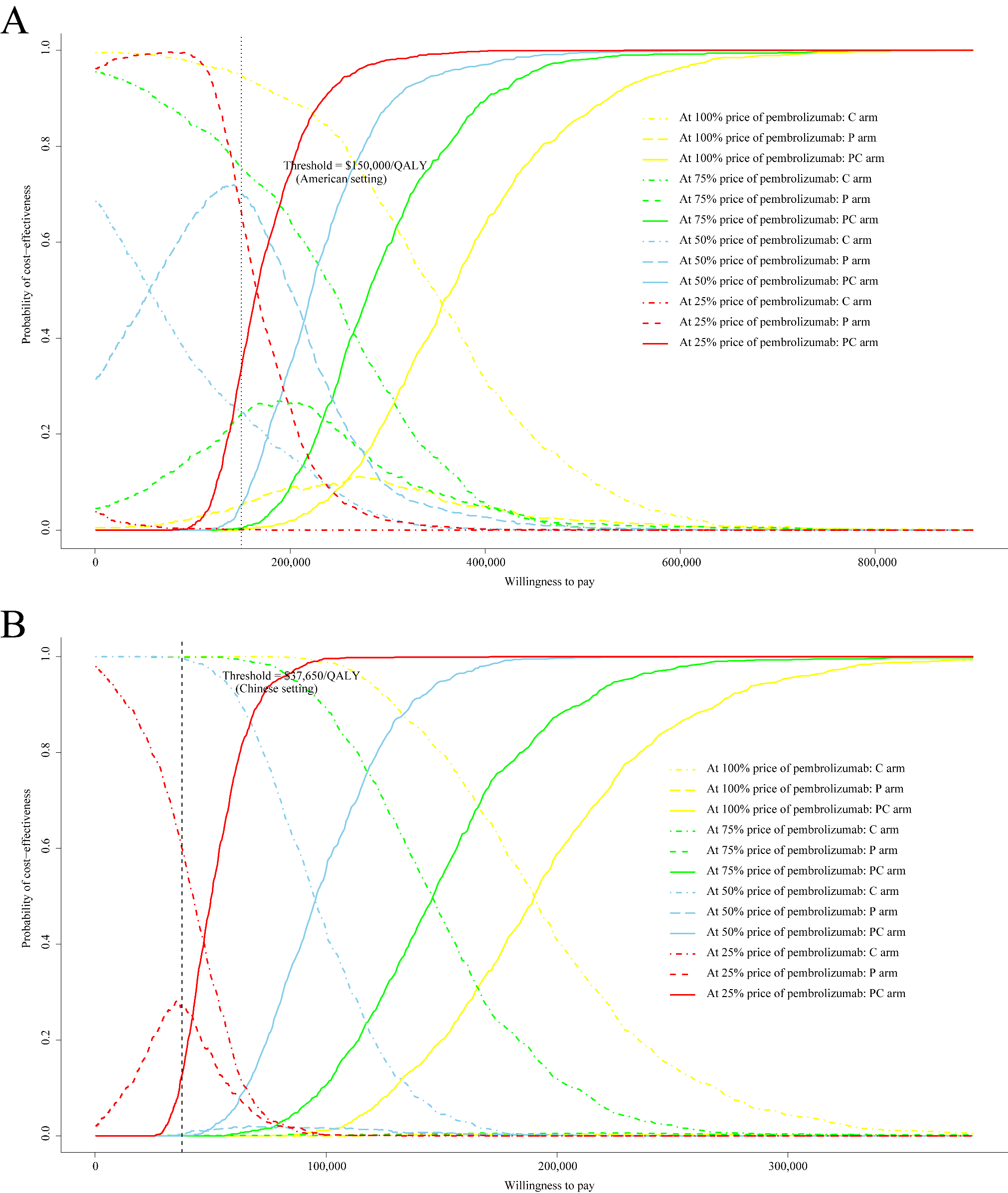


Supplemental Figure 3. Cost-effectiveness acceptable curve. (A)The output in the American setting. (B) The output in the Chinese setting. The y-axis indicates the probability that a regimen is cost-effective across the willingness-to-pay threshold (x-axis). ***QALY*** quality-adjusted life-year, ***PC*** pembrolizumab plus chemotherapy, ***P*** pembrolizumab, ***C*** chemotherapy.

| Supplemental Table 2. Results of the Probabilistic sensitivity analysis in several scenarios | | | | | |
| --- | --- | --- | --- | --- | --- |
| Country | **Regimen** | **At the WTP threshold, the likelihood of cost-effective when the price of the pembrolizumab was__________** | | | |
|  |  | **the base-value** | **75% of the base-value** | **50% of the base-value** | **25% of the base-value** |
| The United States | chemotherapy | 94.8% | 75.3% | 24.8% | 0.1% |
|  | pembrolizumab | 5.1% | 24.3% | 69.9% | 66.1% |
|  | pembrolizumab plus chemotherapy | 0.1% | 0.4% | 5.3% | 33.8% |
| China | chemotherapy | 100% | 99.9% | 99.7% | 60% |
|  | pembrolizumab | 0% | 0.1% | 0.3% | 27.3% |
|  | pembrolizumab plus chemotherapy | 0% | 0% | 0.0% | 12.7% |
| *WTP* willingness to pay. | | | | | |
